# Chlorination of arenes via the degradation of toxic chlorophenols

**DOI:** 10.1073/pnas.2122425119

**Published:** 2022-05-19

**Authors:** Mingyang Liu, Xuemei Yang, Paul J. Dyson

**Affiliations:** ^a^Institute of Chemical Sciences and Engineering, Ecole Polytechnique Fédérale de Lausanne (EPFL), 1015 Lausanne, Switzerland

**Keywords:** chlorophenol pollutant, aryl chloride, isofunctional reaction, decontamination

## Abstract

Aryl chlorides are among the most versatile synthetic precursors, and yet inexpensive and benign chlorination techniques to produce them are underdeveloped. We propose a process to generate aryl chlorides by chloro-group transfer from chlorophenol pollutants to arenes during their mineralization, catalyzed by Cu(NO_3_)_2_/NaNO_3_ under aerobic conditions. A wide range of arene substrates have been chlorinated using this process. Mechanistic studies show that the Cu catalyst acts in cooperation with NO_x_ species generated from the decomposition of NaNO_3_ to regulate the formation of chlorine radicals that mediate the chlorination of arenes together with the mineralization of chlorophenol. The selective formation of aryl chlorides with the concomitant degradation of toxic chlorophenol pollutants represents a new approach in environmental pollutant detoxication. A reduction in the use of traditional chlorination reagents provides another (indirect) benefit of this procedure.

## Significance

Chlorination reactions are widely applied in organic synthesis, with aryl chlorides being key intermediates in the synthesis of many pharmaceutical products. Here, we demonstrate that waste materials such as chlorophenol pollutants can be valorized as chlorination reagents via catalytic transfer of the chloro group during their mineralization for the generation of valuable aryl chlorides. This process adds value to the destruction of chlorophenol pollutants, and the concept could potentially be extended to the valorization of other classes of stockpiles awaiting mineralization.

**Fig. 1.**
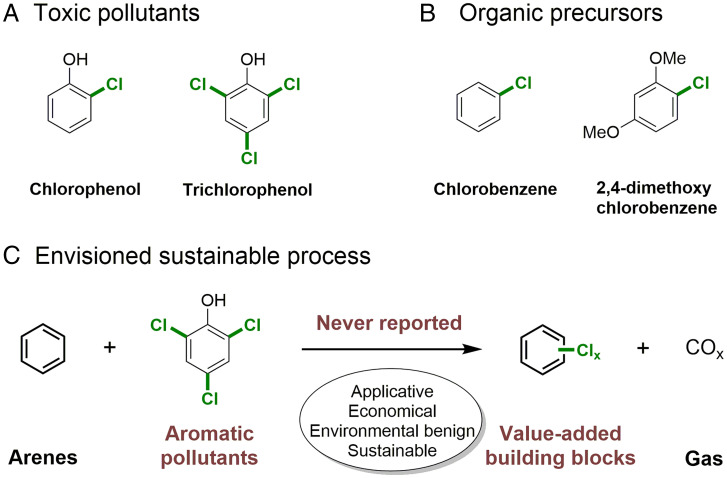
Background and reaction design. (*A*) Examples of chlorophenol pollutants. (*B*) Examples of aryl chlorides. (*C*) The chlorination process reported herein was based on chloro-group transfer from chlorophenol pollutants.

Chlorophenols are widely encountered moieties present in herbicides, drugs, and pesticides (1). Owing to the high dissociation energies of carbon‒chloride bonds, chlorophenols biodegrade very slowly, and their prolonged exposure leads to severe ecological and environmental problems (Fig. 1*A*) (2–4). Several well-established technologies have been developed for the treating of chlorophenols, including catalytic oxidation (5–11), biodegradation (12–15), solvent extraction (16, 17), and adsorption (18–20) Among these methods, adsorption is the most versatile and widely used method due to its high removal efficiency and simple operation, but the resulting products are of no value, and consequently, these processes are not viable.

With the extensive application of substitution reactions (21, 22), transfunctionalizations (23, 24), and cross-coupling reactions (25, 26), aryl chlorides are regarded as one of the most important building blocks widely used in the manufacture of polymers, pharmaceuticals, and other types of chemicals and materials (Fig. 1*B*) (27–31). Chlorination of arenes is usually carried out with toxic and corrosive reagents (32–34). Less toxic and more selective chlorination reagents tend to be expensive [e.g., chloroamides (35, 36)] and are not atom economic (37–39). Consequently, from the perspective of sustainability, the ability to transfer a chloro group from unwanted chlorophenols to other substrates would be advantageous.

Catalytic isofunctional reactions, including transfer hydrogenation and alkene metathesis, have been widely exploited in organic synthesis. We hypothesized that chlorination of arenes also could be achieved by chloro-group transfer, and since stockpiles of chlorophenols tend to be destroyed by mineralization and chlorophenol pollutants may be concentrated by adsorption (18–20), they could be valorized as chlorination reagents via transfer of the chloro group to arene substrates during their mineralization, thereby adding value to the destruction process (Fig. 1*C*). Although chlorophenol pollutants are not benign, their application as chlorination reagents, with their concomitant destruction to harmless compounds, may be considered as not only meeting the criteria of green chemistry but also potentially surpassing it. Herein, we describe a robust strategy to realize chloro-group transfer from chlorophenol pollutants to arenes and afford a wide range of value-added aryl chlorides.

## Results and Discussion

**Fig. 2.**
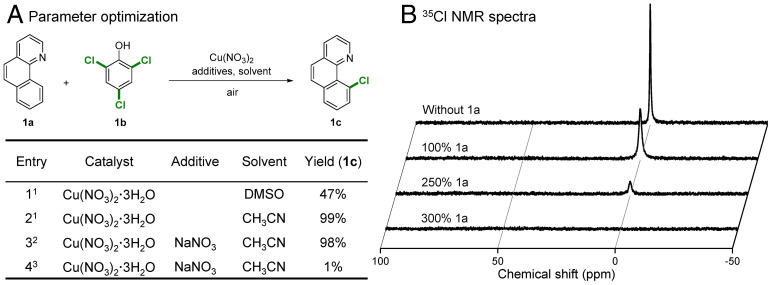
Reaction optimization and results. (*A*) Parameter optimization. Reaction conditions: 1) Cu(NO_3_)_2_·3H_2_O (60 mol%, 0.18 mmol), 7,8-benzoquinoline (0.3 mmol), 2,4,6-trichlorophenol (0.1 mmol), solvent (2 mL), biphenyl (0.1 mmol) as internal standard, air (0.5 MPa), 140 °C, 10 h; 2) Cu(NO_3_)_2_·3H_2_O (20 mol%, 0.06 mmol), NaNO_3_ (50 mol%, 0.15 mmol); 3) Ar atmosphere. Yield was determined by GC. (B) ^35^Cl NMR spectra showing the evolution of chloride ions as a function of the substrate concentration. Reaction conditions were the same as 2), but with different amounts of **1a** as indicated in the figure.

Our initial studies focused on the chlorination of the model compound 7,8-benzoquinoline (**1a**), since it contains both an arene ring and a heterocycle, using 2,4,6-trichlorophenol (**1b**) as a chlorination reagent in dimethyl sulfoxide (DMSO). Various Cu salts were evaluated as catalysts (*SI Appendix*, Table S1), with 60 mol% Cu(NO_3_)_2_ affording 10-chlorobenzo[h]quinoline (**1c**) in moderate yield (47%) (Fig. 2*A*, entry 1). Changing the solvent to CH_3_CN considerably improves the yield of the chlorination reaction, with **1c** obtained in near-quantitative yield (99%; Fig. 2*A*, entry 2). The reaction conditions were further optimized, allowing the catalyst loading to be decreased to 20 mol% [i.e., Cu(NO_3_)_2_ together with NaNO_3_ additive]. If 20 mol% of Cu(NO_3_)_2_ without NaNO_3_ or alternatively pure O_2_ is used as oxidant, the yield of the reaction decreases to 60 to 62% (*SI Appendix*, Table S3, entry 9 to 10). Previous studies indicate that the nitrate anion is an efficient catalyst (40, 41) or cocatalyst (42, 43) in redox reactions. As expected, the combination of Cu(NO_3_)_2_ and NaNO_3_ affords **1c** in near-quantitative yield (98%; Fig. 2*A*, entry 3). Notably, the reaction was inhibited when conducted under an argon atmosphere (Fig. 2*A*, entry 4). Further details of the optimization of the reaction conditions are provided in *SI Appendix*, Tables S1–S5.

^35^Cl NMR spectroscopy was used to monitor the reaction in CD_3_CN (Fig. 2*B*). In the absence of a substrate, 2,4,6-trichlorophenol (**1b**) reacts to qualitatively form Cl^−^ (determined by comparison with the ^35^Cl NMR spectra of a standard solution containing NaCl). In the presence of the model substrate (**1a**), the peaks corresponding to the chloride ions in the ^35^Cl NMR spectra gradually decreased (Fig. 2*B*). The spectra show that the chloride generated is fully transferred to the substrate to generate **1c**. ^1^H, ^13^C- Heteronuclear Single Quantum Coherence (HSQC) NMR spectroscopy was used to study the evolution of organic species (i.e., the consumption of **1a** and **1b** and concomitant formation of **1c**) (*SI Appendix*, Fig. S1). The characteristic contours (black) of target reaction site (signal 9 in *SI Appendix*, Fig. S1) in **1a** and characteristic signal (red) of **1b** were observed before reaction. After reaction, characteristic contours of **1a** and **1b** completely disappeared and transformed into signals attributable to the product (**1c**, green contours). Notably, signals corresponding to other aromatics and other species were not detected in the liquid phase. Volatile products were also collected and analyzed by gas chromatography (GC)–mass spectrometry (MS) with both CO and CO_2_ detected (*SI Appendix*, Fig. S2). Combined, these studies demonstrate the high conversion and selectivity of this unique chlorination reaction.

### Mechanistic Studies.

**Fig. 3.**
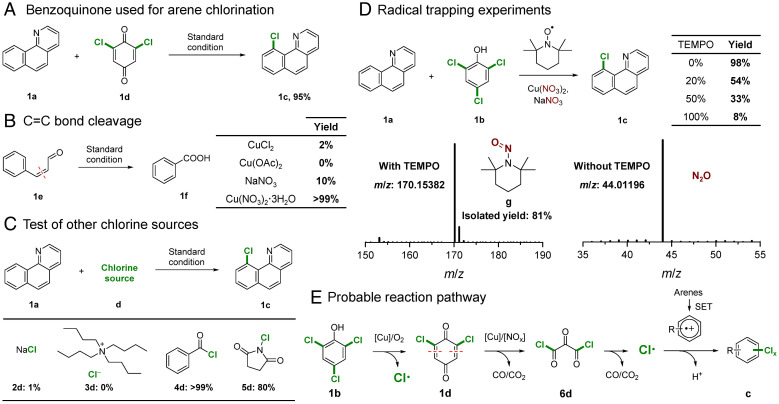
Mechanistic studies. (*A*) Application of benzoquinone as a chlorination reagent. (*B*) Using cinnamaldehyde **1e** as a substrate. (*C*) Evaluation of other chlorination sources. (*D*) Radical trapping experiments employing TEMPO under the standard reaction conditions. (*E*) Proposed reaction pathway. R = pyridine, pyrimidine, or methoxy. Standard reaction conditions: Cu(NO_3_)_2_·3H_2_O (20 mol%, 0.06 mmol), NaNO_3_ (50 mol%, 0.15 mmol), substrate (0.3 mmol), chlorination reagent (100 mol%), CH_3_CN (2 mL), biphenyl (0.1 mmol) as internal standard, air (0.5 MPa), 140 °C, 10 h.

It is known that phenols are readily transformed into benzoquinone derivatives under Cu-catalyzed oxidative conditions (44, 45). Thus, employing chlorobenzoquinone (**1d**) as a chlorination reagent gave the expected product **1c** in excellent yield (95%; Fig. 3*A*), implying a benzoquinone-mediated pathway. Benzoquinone **1d** was not observed during the chlorination reaction, but based on the literature report (44, 45) and the higher reaction rate observed using chlorobenzoquinone compared to chlorophenol, **1d** is presumably an intermediate with the dechlorination of **1b** to **1d** being the rate-determining step (*SI Appendix*, Fig. S3).

To verify whether the system can oxidatively cleave C = C bonds, cinnamaldehyde (**1e**) was employed as a starting material together with various Cu catalysts (Fig. 3*B*). CuCl_2_ and Cu(OAc)_2_ salts as well as NaNO_3_ used as a control displayed low activity in the oxidative cleavage of the C = C bonds and the formation of benzoic acid (**1f**). In contrast, the combination of Cu and nitrate ions [i.e., Cu(NO_3_)_2_] displayed high activity in this reaction to afford **1f** in high yield (86%), indicating synergy between the Cu and nitrate ions in cleaving C = C bonds and achieving ring opening of benzoquinone to promote chlorination.

Other potential chlorination reagents were also investigated (Fig. 3*C*). Ionic chloride sources, including NaCl (**2d**) and tetrabutylammonium chloride (**3d**), were inactive for the chlorination of **1a**, whereas organochlorides such as benzoyl chloride (**4d**) and *N*-chlorosuccinimide (**5d**) were effective. These organochlorides have similar characteristics that are conducive to generating Cl·radicals (46, 47), suggesting that the chlorination reaction proceeds via a radical-mediated pathway. Cl radicals are therefore considered to be the active chlorination reagent and not Cl^−^ ions. However, if Cl radicals were not captured by arenes, Cl radicals would reduce to Cl anions, which were detected by ^35^Cl NMR spectroscopy in the absence of an arene substrate (Fig. 2*B* and *SI Appendix*, Fig. S4). Hence, no reservoir of Cl is needed as the Cl radicals react as soon as they are formed.

To further probe the possibility of a radical-mediated reaction mechanism, radical trapping experiments were performed by introducing (2,2,6,6-tetramethylpiperidin-1-yl)oxyl (TEMPO) into the reaction under the standard conditions (Fig. 3*D*). Stoichiometric amounts of TEMPO largely suppress the chlorination of **1a**, with **1c** obtained in only 8% yield. NO radicals were trapped by TEMPO to give 2,2,6,6-tetramethyl-1-nitrosopiperidine (**g**) (Fig. 3*D* and *SI Appendix*, Figs. S5 and S6). Moreover, N_2_O was detected and identified in the standard reaction in the absence of TEMPO (typical reaction; *SI Appendix*, Fig. S2), indicating that a redox reaction between NO and N_2_O under aerobic condition plays a key role during the radical-mediated chlorination reaction. NO_x_ species are obtained from the thermal decomposition of the nitrate salt (41), rationalizing the observed solvent effect of the reaction (Fig. 2*A* and *SI Appendix*, Fig. S7). The gaseous products obtained from heating Cu(NO_3_)_2_ in different solvents were collected and analyzed (*SI Appendix*, Fig. S7). N_2_O is the main gas obtained in CH_3_CN, confirming the key role of N_2_O in the reaction. These findings are in good agreement with a previous study that showed NO_x_ species generated from nitrate salts may act as cocatalysts (42, 48). We attempted to capture NO radicals generated from the catalyst and/or Cl radicals generated from the substrates by adding radical trapping agent 5,5-dimethyl-1-pyrroline *N*-oxide (DMPO) into the reaction mixture (Fig. 3*A*, entry 3) to form a stable DMPO–radical complex and employed electron paramagnetic resonance (EPR) spectroscopy. However, the EPR spectra did not provide clear evidence for the presence of the Cl radical due to masking by the strong EPR signals from the Cu(II) catalyst (*SI Appendix*, Fig. S8) (49, 50).

Based on these experiments, we tentatively propose that the chlorination pathway with chlorophenols involves two main parts: the decomposition of phenol to generate the Cl radical catalyzed by Cu(NO_3_)_2_/NO_x_ and the Cu-catalyzed radical-mediated chlorination of arenes (51, 52). A plausible mechanism was proposed (Fig. 3*E*). In the first step, 2,4,6-trichlorophenol (**1b**) undergoes Cu-catalyzed oxidation to afford 2,6-dichlorobenzoquinone (**1d**) together with the release of a Cl radical (*SI Appendix*, Fig. S9). Next, ring opening through oxidative C = C bond cleavage catalyzed by Cu/NO_x_ affords the acyl chloride derivative (**6d**) (*SI Appendix*, Figs. S10 and S11), which decomposes to release two Cl radicals. In the final step, the Cl radicals generated during the mineralization of **1b** undergo Cu-catalyzed radical-type chlorination to produce aryl chloride products (**c**) (*SI Appendix*, Fig. S12 and Table S6) (51–56). Further details concerning each step of the reaction mechanism are provided in the *SI Appendix*.

### Substrate and Reagent Scope.

**Fig. 4.**
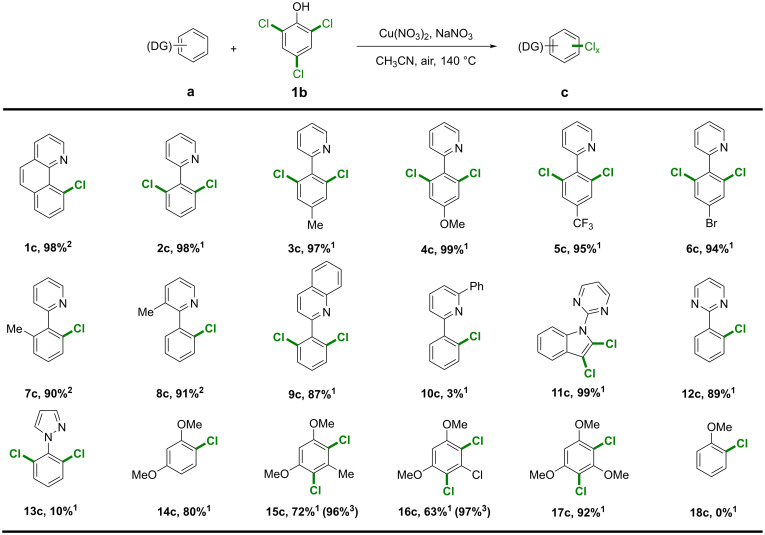
Substrate scope. Reaction conditions: 1) Cu(NO_3_)_2_·3H_2_O (0.06 mmol), NaNO_3_ (0.15 mmol), arene (0.3 mmol), 2,4,6-trichlorophenol (0.2 mmol), CH_3_CN (2 mL), biphenyl (0.1 mmol) as internal standard, air (0.5 MPa), 140 °C, 10 h; 2) 2,4,6-trichlorophenol (0.1 mmol); 3) 24 h. Yield was determined by GC. The amounts of chlorophenol and arene substrate employed were calculated based on the number of Cl groups on the chlorophenol reagent and the aryl chloride product. The molar ratio of Cl used was 1:1. Therefore, the high yield of aryl chloride products (>90%) indicated almost full transfer of the Cl groups from the chlorination reagent to the product. DG: directing group.

To establish the synthetic utility of the chlorination reaction, we extended the reaction to other substrates (Fig. 4). Various aromatic derivatives with pyridyl as a directing group were tested under standard reaction conditions. Substituent groups at the *para* position had little effect and gave highly regioselective aryl *ortho*-dichloride products in excellent yields (**2c**-**6c**). Hindered substrates [i.e., 2-arylpyridines with a methyl group at the *ortho*-position of the phenyl ring (**7a**) or pyridyl (**8a**) ring] were transformed into *ortho*-monochloride products. Replacing the pyridyl ring with a quinolyl system (**9a**) also afforded the corresponding *ortho*-monochloride product (**9c**). The transformation of symmetric diphenyl pyridine (**10a**) was problematic, affording the monochloride in only very low yield due to the formation of a stable Cu-coordinated intermediate identified by MS (*SI Appendix*, Fig. S13). Substrates with other types of directing groups, including a pyrimidine-substituted heterocycle (**11**-**13a**), *N*-(2-pyrimidyl)indole (**11a**), or 2-phenylpyrimidine (**12a**), were also successfully transformed in high yield. However, the pyrazole group (**13a**) was found to be strongly deactivating, with the desired product obtained in low yield.

The chlorination reaction was successfully expanded to electron-rich substrates without directing groups (**14a**-**18a**), although in some cases prolongation of the reaction time was required. 1,3-Dimethoxybenzene (**14a**) gave the expected monochloride product in 80% yield, and the reaction of several substituted 1,3-dimethoxybenzene compounds (**15**-**17a**) afforded highly regioselective dichloride products in excellent yields. Unfortunately, with simple arenes such as benzene and toluene, the aromatic ring is extremely stable, preventing oxidation without the addition of radical initiators (54, 55).

The chlorination reaction may be applied to arenes substituted with *N*-containing (pyridine or pyrimidine) groups or multiple methoxy groups, which are crucial for the activation of the benzene ring to generate the reactive cation-radical intermediate, which captures the Cl radical obtained from the mineralization of the chlorophenol reagent (*SI Appendix*, Fig. S12) (51–56). The *N*-containing groups and methoxy groups are both directing groups but play different mechanistic roles: The former directs chlorination via coordination to the Cu catalyst, whereas the latter acts as electron-donating groups directing the position of electrophilic substitution. Chlorination of anisole with a single methoxy group (**18a**) does not proceed, excluding the directing role via coordination.

**Fig. 5.**
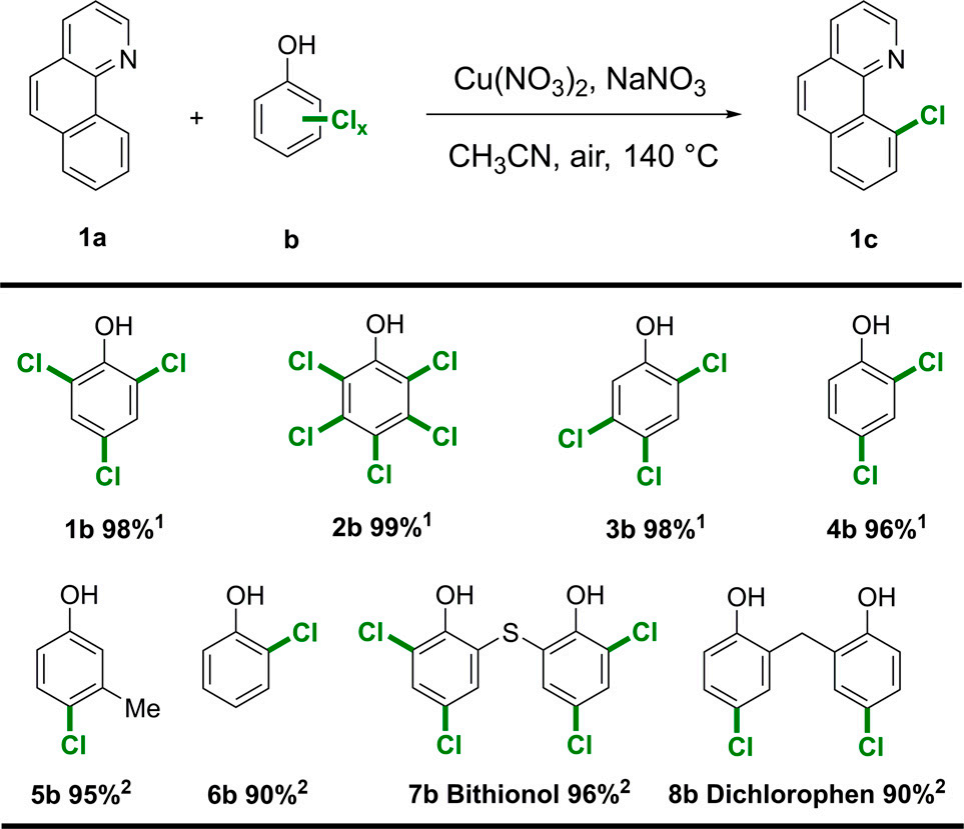
Scope of chlorophenol reagents. Reaction conditions: 1) Cu(NO_3_)_2_·3H_2_O (0.06 mmol), NaNO_3_ (0.15 mmol), **1a** (0.3 mmol), 100 mol% chlorophenol based on the Cl-content, CH_3_CN (2 mL), biphenyl (0.1 mmol) as internal standard, air (0.5 MPa), 140 °C, 10 h; 2) 24 h. Yield was determined by GC.

Chlorinated phenols, which contain only an aromatic skeleton with chloro substituents, are among the most hazardous pollutants, as they are highly toxic and persist in nature (57, 58). Hence, several chlorinated phenol monomers (**1b**-**6b**) were employed as chlorination reagents employing **1a** as the substrate, and all produced the expected product **1c** in excellent yield (90 to 99%) (Fig. 5). Specifically, monochlorinated phenols (**5a**, **6a**) result in a lower activity and require longer reaction times in order to achieve satisfactory yields. Chlorinated phenol dimers are typically more toxic and more resistant to degradation, but by increasing the reaction time, bithionol (**7b**) and dichlorophen (**8b**) were successfully used for the chlorination of **1a**, affording **1c** in >90% yield.

## Conclusions

The production of aryl chlorides was achieved using chloro-group transfer from chlorophenol pollutants during their destruction to arene substrates, thus valorizing the Cl atoms within them with a very high atom economy. The Cu(NO_3_)_2_ catalyst and NaNO_3_ cocatalyst are inexpensive, abundant, and nontoxic, and only gaseous degradation by-products are produced. A broad range of substrates can be transformed that contain a range of different directing groups with both electron-rich and electron-poor substituents. Moreover, various kinds of chlorophenol pollutants may be used as the source of chlorine. We anticipate that using chlorophenols in synthesis will lead to the utilization of other chemical waste that would otherwise be mineralized.

## Materials and Methods

### Materials.

Pyridyl-based substrates were purchased from Fluorochem. Chlorophenols and Cu salts were purchased from Sigma-Aldrich. Solvents including acetonitrile, ethyl acetate, dimethyl sulfoxide, and dimethyl formamide were purchased from Acros. Additives and other chemicals, including NaNO_3_, biphenyl, tetrabutylammonium chloride, 2,2,6,6-tetramethyl-1-nitrosopiperidine, and 1,1-diphenylethylene were purchased from Sigma-Aldrich, Acros, or Alfa Aesar. All chemicals were used as received without further purification.

### Characterization.

Qualitative and quantitative analysis of liquid samples was performed by GC, Agilent 7890B, equipped with a mass detector (Agilent 7000C) and hydrogen flame-ionization detector (FID) HP-5 polar column. GC yield was determined based on internal standard curves and areas of integrated peak area. Qualitative analysis of gas samples was performed on a Thermo Fisher Scientific TSQ8000 Triple Quadrupole GC-MS/MS instrument equipped with packed HayeSep Q 80/100 columns and a thermal conductivity detector (TCD). Quantitative analysis of gas samples was performed on an Agilent 7890B equipped with packed HayeSep Q 80/100 columns, an FID, and a TCD. Yields were determined using an external standard method based on pure standard gases.

^1^H, ^13^C, ^35^Cl, and ^1^H,^13^C- HSQC NMR spectra were recorded on a Bruker Avance III HD 400 instrument equipped with a 5-mm BBFO probe. CD_3_CN, DMSO-d_6_, or CDCl_3_ was used as solvent. The resonance band of tetramethylsilane or solvent was used as the internal standard. Prior to recording ^35^Cl and HSQC NMR spectra, a stoichiometric amount of NaBH_4_ was added to the reaction mixture to remove the Cu salt, and the filtered reaction mixture was used for ^35^Cl NMR experiments.

EPR spectra were recorded on a Bruker EMXnano instrument (high-performance bench-top EPR system). Microwave frequency was 9.60 GHz (X band). Low-temperature EPR measurements were performed at 100 K. High boiling benzonitrile was used as the solvent instead of acetonitrile so the reaction could be conducted at atmospheric pressure rather than in an autoclave. After reaction for 4 h, the reaction mixture (0.2 mL) was mixed with DMPO (0.2 mL, 0.5 mmol/mL), and the mixture was cooled with liquid N_2_ prior to measurements.

### General Procedure for the Chlorination Reaction.

Substrate (0.3 mmol), 2,4,6-trichlorophenol (0.1 mmol, 100 mol% chlorophenol based on Cl), Cu(NO_3_)_2_·3H_2_O (0.06 mmol), NaNO_3_ (0.15 mmol), CH_3_CN (2 mL), and biphenyl (0.1 mmol) as internal standard were added into a stainless steel reactor with a quartz liner. After charging with compressed air (0.5 MPa), the reactor was heated at 140 °C for 10 h. After reaction, the gas phase was collected in a Tedlar Push Lock Valve gas sampling bag. Ethyl acetate and saturated aqueous NH_4_Cl was added into the liquid mixture to separate the products. The organic matter was extracted with ethyl acetate twice. Further purification was conducted by gel column chromatography.

## Supplementary Material

Supplementary File

## Data Availability

All study data are included in the article and/or *SI Appendix*.
