# Supplementary material for: Chlorination of arenes via the degradation of toxic chlorophenols
Source: Proc Natl Acad Sci U S A. 2022 May 19;119(21):e2122425119. doi: 10.1073/pnas.2122425119 (PMC9173806; doi:10.1073/pnas.2122425119)
Supplement: Supplementary File [file pnas.2122425119.sapp.pdf]

Chlorination of Arenes via the Degradation of Toxic Chlorophenols

Mingyang Liu, Xuemei Yang and Paul J. Dyson*

Institute of Chemical Sciences and Engineering, Ecole Polytechnique Fédérale de Lausanne (EPFL), 1015 Lausanne, Switzerland.

Table of Contents

Experimental details 2

Supplementary results and discussion 4

References 38

NMR spectra 16

Experimental details

Materials

Pyridyl-based substrates were purchased from Fluorochem. Chlorophenols and Cu salts were purchased from Sigma-Aldrich. Solvents including acetonitrile, ethyl acetate, dimethylsulfoxide, and dimethyl formamide were purchased from Acros. Additives and other chemicals including $NaNO_3$, biphenyl, tetrabutylammonium chloride, 2,2,6,6-tetramethyl-1-nitrosopiperidine, 1,1-diphenylethylene were purchased from Sigma-Aldrich, Acros or Alfa Aesar. All chemicals were used as received without further purification.

Characterization

Qualitative and quantitative analysis of liquid samples was performed by gas chromatography (GC), Agilent 7890B, equipped with mass detector (Agilent 7000C) and hydrogen flame-ionization detector (FID), HP-5 polar column. GC yield was determined based on internal standard curves and areas of integrated peak area. Qualitative analysis of gas samples was performed on Thermo scientific TSQ8000 Triple Quadrupole GC-MS/MS instrument equipped with packed HayeSep Q 80/100 columns and a thermal conductivity detector (TCD). Quantitative analysis of gas samples was performed on an Agilent 7890B equipped with packed HayeSep Q 80/100 columns, FID and TCD detector. Yields were determined using an external standard method based on pure standard gases.

^{1}H, ^{13}C, ^{35}Cl and ^{1}H,^{13}C-Heteronuclear Single Quantum Coherence (HSQC) NMR spectra were recorded on a Bruker Avance III HD 400 instrument equipped with a 5 mm BBFO probe. CD_3CN, DMSO-d_6 or $CDCl_3$ were used as solvent. The resonance band of tetramethylsilane (TMS) or solvent was used as the internal standard. Prior to recording ^{35}Cl and HSQC NMR spectra, a stoichiometric amount of $NaBH_4$ was added to the reaction mixture to remove the Cu salt and the filtered reaction mixture was used for ^{35}Cl NMR experiments.

Electron paramagnetic resonance (EPR) spectra were recorded on Bruker EMXnano instrument (high-performance bench-top EPR system). Microwave frequency is 9.60 GHz (X band). Low-temperature EPR measurements were performed at 100 K. High boiling benzonitrile was used as the solvent instead of acetonitrile in order that the reaction could be conducted at atmospheric pressure rather than in an autoclave. After reaction for 4 h, the reaction mixture (0.2 mL) was mixed with 5,5-Dimethyl-1-pyrroline N-oxide (DMPO, 0.2 mL, 0.5 mmol/mL) and the mixture was cooled with liquid N_2 prior to measurements.

Synthesis of N-(2-pyrimidyl)indole **11a**[1]

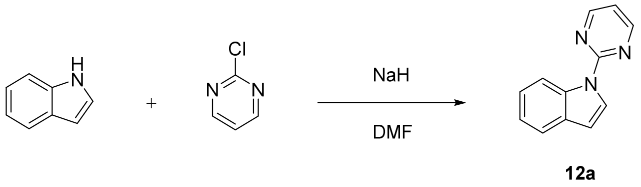

Indole (20 mmol) was dissolved in 50 mL DMF under a N_2 atmosphere. NaH (30 mmol, 60% dispersion in mineral oil) was added slowly. The reaction mixture was stirred at room temperature for 30 min and 30 mmol 2-chloropyrimidine was added. The reaction was stirred at 150 °C for 16 h until the indole was completely consumed. The resulting reaction was quenched with a saturated aqueous NH_4Cl solution. The organic compounds were extracted with ethyl acetate (3 times), washed with a saturated aqueous NH_4Cl solution, brine, water, and dried with sodium sulfate. Concentration in vacuo and purification using silica gel chromatography of the crude product gave **11a** as a white powder.

Synthesis of 2-phenylpyrimidine **12a**[2]

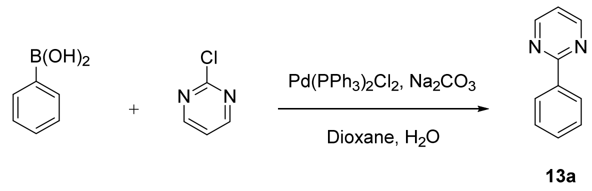

Phenylboronic acid (14.4 mmol), 2-chloropyrimidine (12 mmol), sodium carbonate (60 mmol), $Pd(PPh_3)_2Cl_2$ (0.15 mmol) were dispersed in dioxane (30 mL) and water (30 mL). The reaction was stirred at 90 °C around 12 h until 2-chloropyrimidine was completely consumed. After reaction, the organic matter was extracted with ethyl acetate for three times, washed with brine, water, and dried with sodium sulfate. Concentration in vacuo and purification by silica gel chromatography gave pure write powder product **12a**.

General procedure for the chlorination reaction

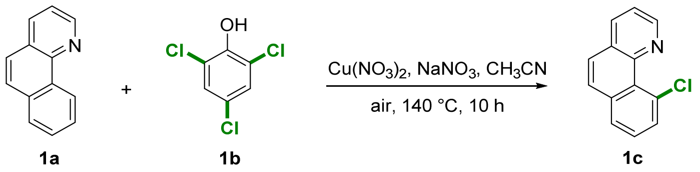

Substrate (0.3 mmol), 2,4,6-trichlorophenol (0.1 mmol, 100 mol% chlorophenol based on Cl), $Cu(NO_3)_2{\cdot}3H_2O$ (0.06 mmol), $NaNO_3$ (0.15 mmol), CH_3CN (2 mL), biphenyl (0.1 mmol) as internal standard were added into stainless-steel reactor with a quartz liner. After charging with compressed air (0.5 MPa), the reactor was heated at 140 °C for 10 h. After reaction, the gas phase was collected in a Tedlar® PLV gas sampling bag. Ethyl acetate and saturated aqueous NH_4Cl was added into the liquid mixture to separate the products. The organic matter was extracted with ethyl acetate twice. Further purification was conducted by gel column chromatography.

Supplementary results and discussion

Table S1. Determination of the optimum Cu catalyst.

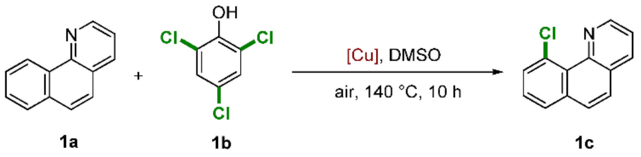

Entry	[Cu]	Yield (**1c**) (%)
1	Cu_2O	0
2	CuO	1
3	Nano CuO	0
4	$Cu(OAc)_2$	0
5	$CuSO_4$	4
6	$Cu(NO_3)_2 \cdot 3H_2O$	47

Reaction conditions: 7,8-benzoquinoline (0.3 mmol), 2,4,6-trichlorophenol (0.1 mmol), Cu salts (0.18 mmol), DMSO (2 mL), biphenyl (0.1 mmol), air (0.5 MPa), 140 ºC, 10 h.

Table S2. Optimization of reaction solvent.

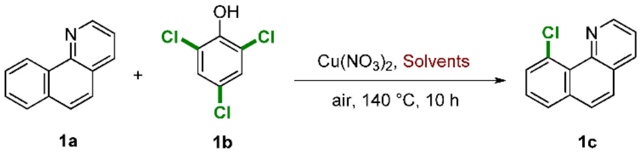

Entry	Solvents	Yield (**1c**) (%)
1	DMSO	47
2	DMF	0
3	Dioxane	21
4	Toluene	66
5	MeOH	26
6	H_2O	12
7	CH_3CN	99

Reaction conditions: 7,8-benzoquinoline (0.3 mmol), 2,4,6-trichlorophenol (0.1 mmol), $Cu(NO_3)_2 \cdot 3H_2O$ (0.18 mmol), solvent (2 mL), biphenyl (0.1 mmol), air (0.5 MPa), 140 ºC, 10 h.

Table S3. Optimization of the additive.[a]

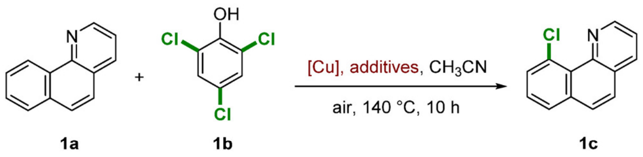

Entry	[Cu]	Additives	Yield (**1c**) (%)
1	$Cu(OAc)_2$	$NaNO_3$	95
2	$CuSO_4$	$NaNO_3$	76
3	$CuCl_2$	$NaNO_3$	92
4	$Cu(NO_3)_2{\cdot}3H_2O$	$NaNO_3$	98
5	$Cu(NO_3)_2{\cdot}3H_2O$	KNO_3	95
6	$Cu(NO_3)_2{\cdot}3H_2O$	NaI	53
7	$Cu(NO_3)_2{\cdot}3H_2O$	30% $NaNO_3$	80
8	$Cu(NO_3)_2{\cdot}3H_2O$	20% $NaNO_3$	75
9	$Cu(NO_3)_2{\cdot}3H_2O$		62
10[b]	$Cu(NO_3)_2{\cdot}3H_2O$		60

Reaction conditions: (a) 7,8-benzoquinoline (0.3 mmol), 2,4,6-trichlorophenol (0.1 mmol), Cu salt (0.06 mmol), additives (0.15 mmol), CH_3CN (2 mL), biphenyl (0.1 mmol), air (0.5 MPa), 140 ºC, 10 h. (b) O_2 (0.5 MPa).

Various Cu salts incorporated with nitrate salts could catalyze the chlorination of **1a** using chlorophenol **1b** as chlorination reagent (Table S3, entries 1-5). Decreasing the $NaNO_3$ ratio leads to a lowering of the product yield (Table S3, entries 7-9).

Table S4. Optimization of the reaction time.

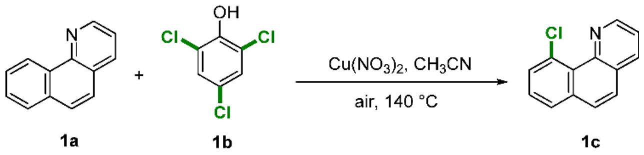

Entry	Reaction time (h)	Yield (**1c**) (%)
1	1	20
2	3	63
3	6	86
4	10	98
5	16	98

Reaction conditions: 7,8-benzoquinoline (0.3 mmol), 2,4,6-trichlorophenol (0.1 mmol), $Cu(NO_3)_2 \cdot 3H_2O$ (0.06 mmol), $NaNO_3$ (0.15 mmol), CH_3CN (2 mL), biphenyl (0.1 mmol), air (0.5 MPa), 140 ºC, 1-16 h.

Table S5. Optimization of reaction temperature.

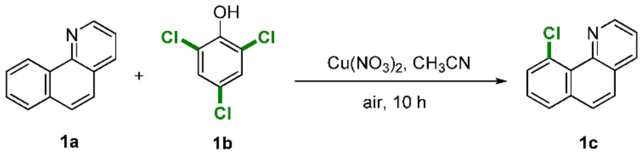

Entry	Temperature (ºC)	Yield (**1c**) (%)
1	100	23
2	120	67
3	130	94
4	140	98
5	150	97

Reaction conditions: 7,8-benzoquinoline (0.3 mmol), 2,4,6-trichlorophenol (0.1 mmol), $Cu(NO_3)_2 \cdot 3H_2O$ (0.06 mmol), $NaNO_3$ (0.15 mmol), CH_3CN (2 mL), biphenyl (0.1 mmol), air (0.5 MPa), 100-150 ºC, 10 h.

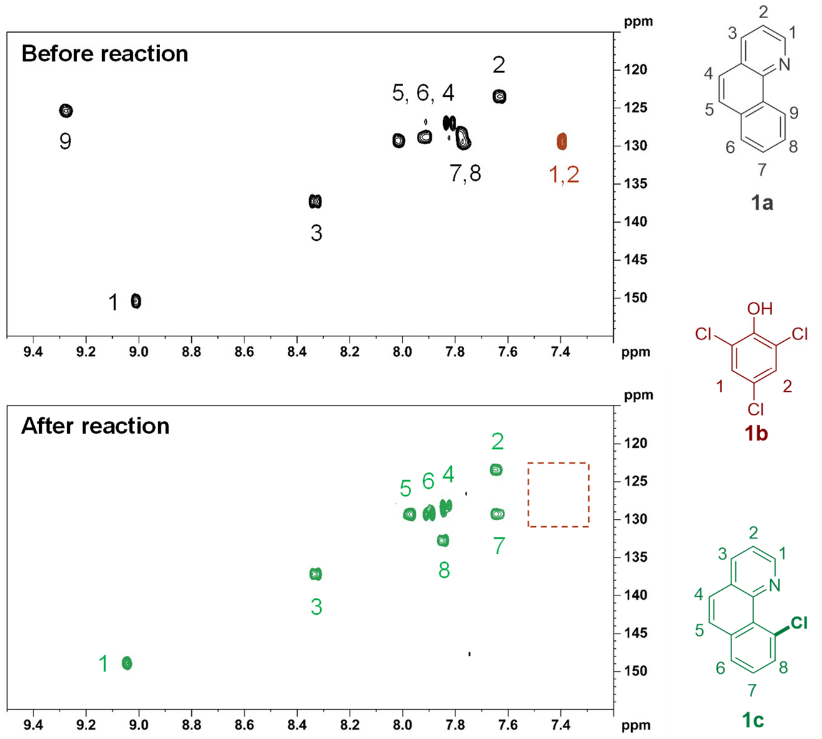

Figure S1. ^{1}H-^{13}C HSQC NMR spectrum of the reaction mixture before and after reaction. Contours are coded to the corresponding structures of substrates and product. Reaction conditions: 7,8-benzoquinoline (**0.5** mmol), 2,4,6-trichlorophenol (**0.1** mmol), $Cu(NO_3)_2{\cdot}3H_2O$ (0.06 mmol), $NaNO_3$ (0.15 mmol), CD_3CN (2 mL), air (0.5 MPa), 140 ºC, 10 h.

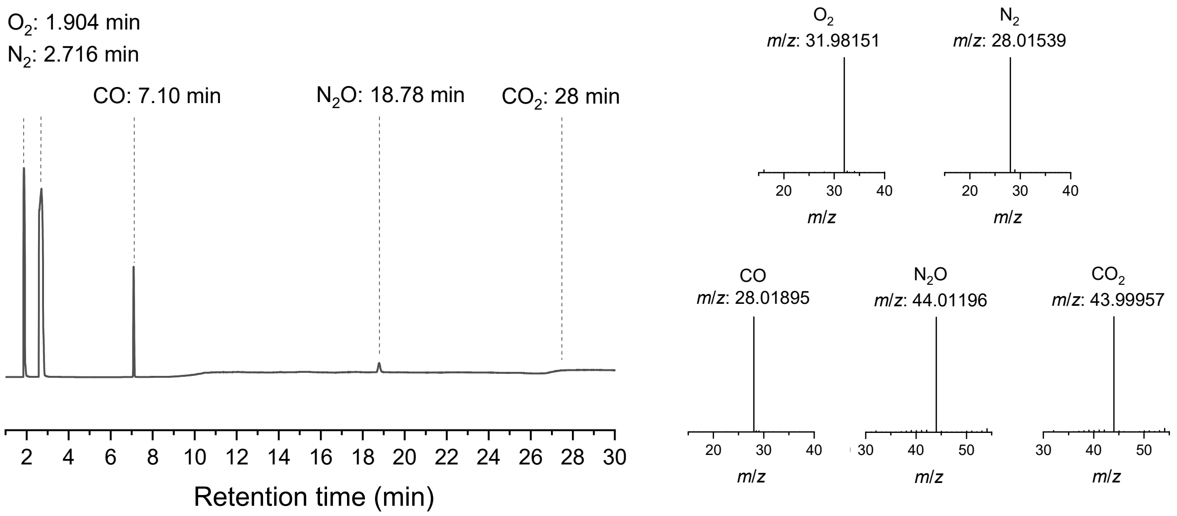

Figure S2. Total ion chromatogram (TIC) and extracted mass spectra of gas products recorded by GC-MS. Reaction conditions were the same as those listed in Figure 2a (reaction conditions 2).

Gas products were collected and analyzed by GC-MS. Qualitative analysis of the gas mixture is based on the extracted mass spectra and a comparison with the appropriate pure standard gas. CO and CO_2 were detected, which were generated from the decomposition of chlorophenol substrates. N_2O was generated from the thermal decomposition of the nitrate salts,[3] and performs an essential role during the chlorination reaction. Further details will be discussed in the part on the mechanistic studies.

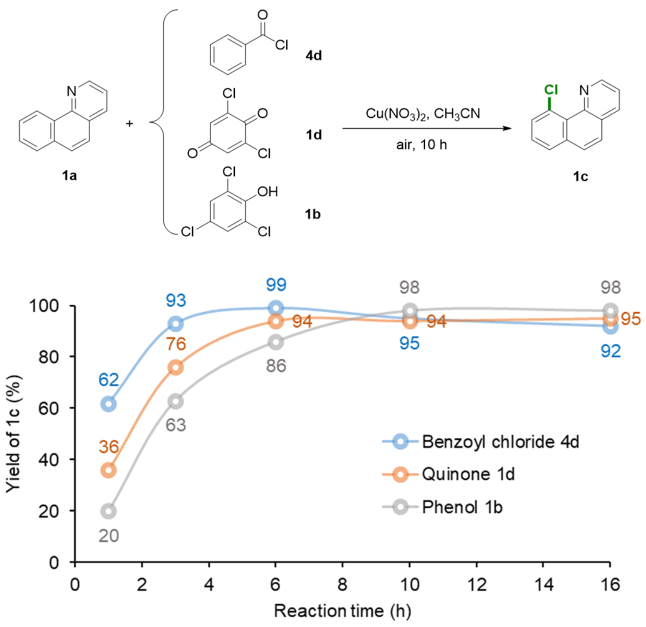

Figure S3. Kinetic study comparing different chlorination reagents using **1a** as substrate. Reaction rate follows the order: **4d** > **1d** > **1b**, indicating that acyl chloride and quinone may be intermediates and the dechlorination of **1b** to **1d** the rate-determining step.

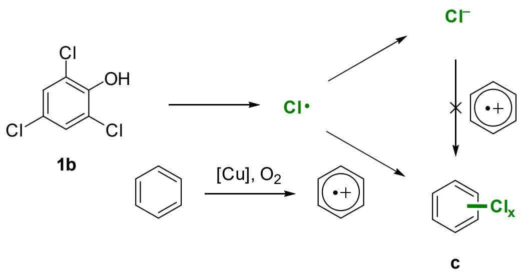

Figure S4. Possible evolution of chlorine species formed during the mineralization of **1b**.

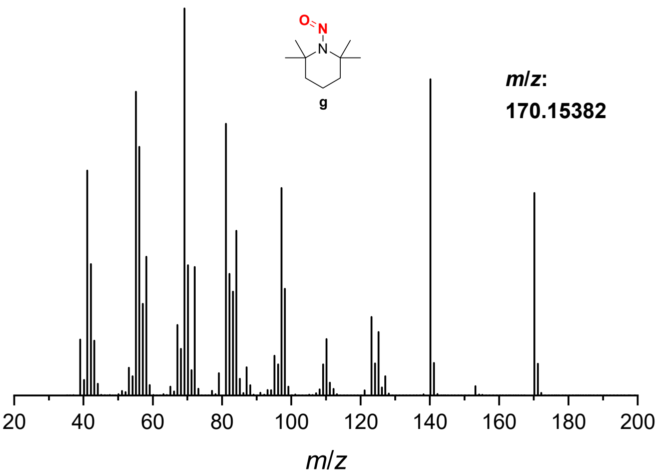

Figure S5. Electron impact ionization (EI) mass spectrum of 2,2,6,6-tetramethyl-1-nitrosopiperidine (**g**).

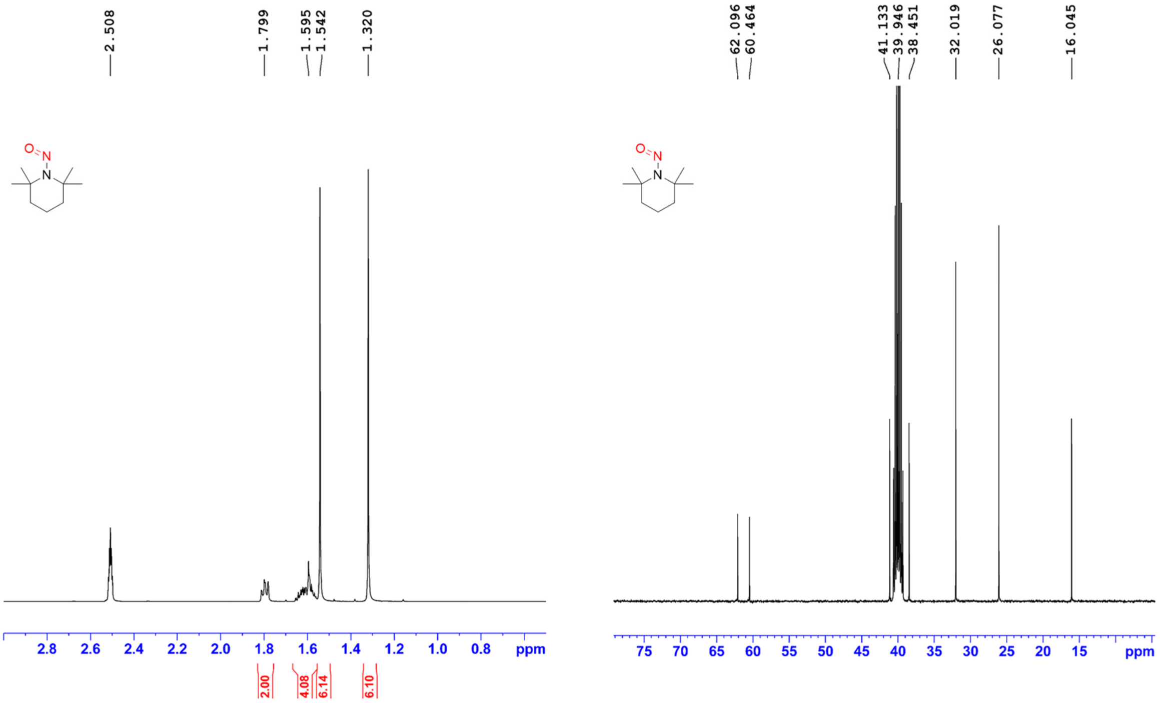

Figure S6. ^{1}H and ^{13}C NMR spectra of 2,2,6,6-tetramethyl-1-nitrosopiperidine (**g**).

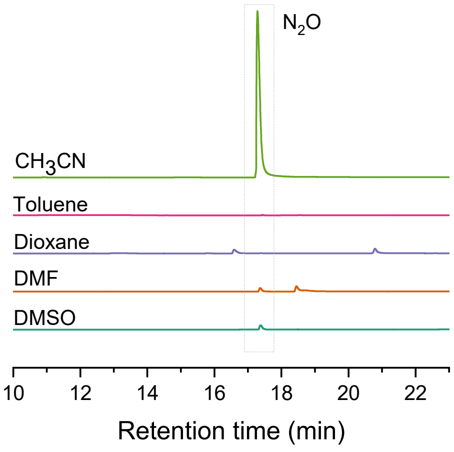

Figure S7. GC spectra of gas products from the decomposition of $Cu(NO_3)_2{\cdot}3H_2O$ in different solvents. Reaction condition: $Cu(NO_3)_2{\cdot}3H_2O$ (0.18 mmol), solvent (2 mL), air (0.5 MPa), 140 ºC, 10 h.

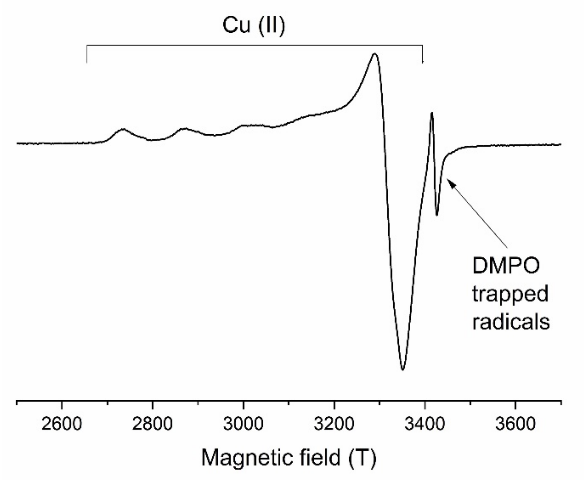

Figure S8. EPR spectrum of the reaction mixture with added DMPO to capture $Cl^{\cdot}$ radicals. Reaction conditions: $Cu(NO_3)_2{\cdot}3H_2O$ (0.06 mmol), $NaNO_3$ (0.15 mmol), **1a** (0.3 mmol), 2,4,6-trichlorophenol (0.1 mmol), benzonitrile (2 mL), exposed in air, 140 ºC, 4 h.

Strong signals from the Cu(II) catalyst are observed ($g_{x,y}$ = 2.066, g_z = 2.327).[4-5] A signal corresponding to DMPO trapped radicals is also present (g = 2.004), but is partially obscured by the strong signals of the Cu(II) catalyst, preventing qualitative analysis of N-, O-, or C-centered radicals.

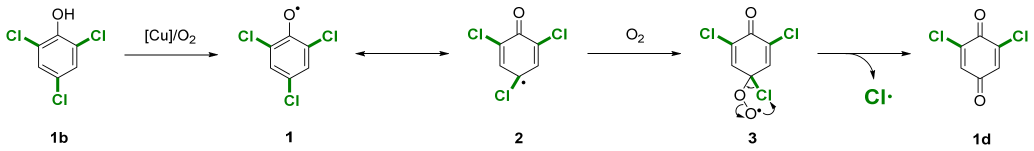

Figure S9. Possible reaction pathway for the Cu catalyzed oxidation of chlorophenol (**1b**) to benzoquinone intermediate (**1d**).

A single electron oxidation of chlorophenol (**1**) catalyzed by the Cu/O_2 system affords phenol radicals **1**. Tautomer **2** of **1** would capture O_2 and activate it to generate the peroxyl radical **3**. After releasing a chlorine radical the benzoquinone intermediate (**2**) would be formed.[6]

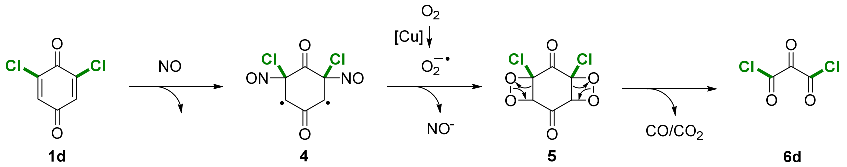

Figure S10. Possible reaction pathway of Cu and NO_x catalyzed oxidative cleavage of the C=C bonds in the benzoquinone intermediate.

NO gas is an active inorganic nitroxyl radical and could attack C=C bonds in benzoquinone to give the C-centered radical intermediate **4**.[3] After capture of a superoxide radical, formed by oxidation in the presence of the Cu catalyst, the dioxetane intermediate **5** would be generated . Further thermal cleavage of **5** would yield the acyl chloride derivative (**6d**). The detailed cycle of the NO_x species is illustrated in Figure S11.

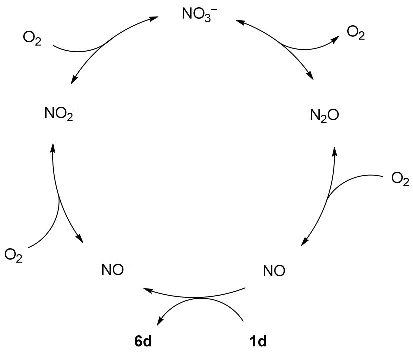

Figure S11. Possible evolution of NO_x species in the aerobic reaction system.

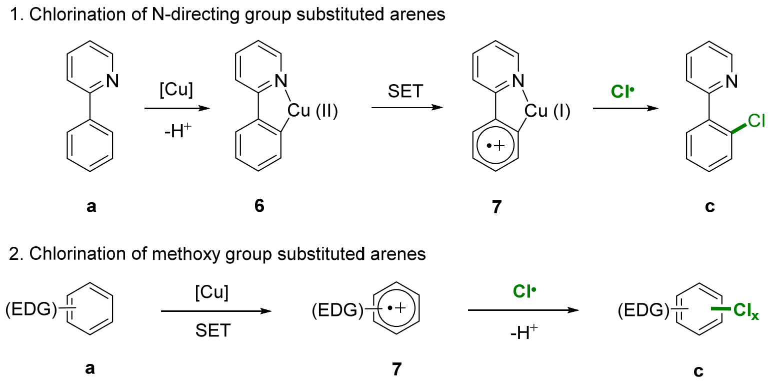

Figure S12. Proposed pathway for the Cu catalyzed radical chlorination reaction.[7-12] SET: single electron transfer. EDG: electron-donating group.

Chlorination of arenes using chlorophenols as chlorination reagents is restricted to arenes with N-containing (pyridine or pyrimidine) groups or electron-donating (methoxy group) groups (Table 1). The Cu catalyzed generation of the cation-radical intermediate **7** appears to be a crucial step for the activation of the arene ring. For arenes with N-containing groups, coordination of the Lewis basic N-atom to the Cu(II) catalyst to form intermediate **6** is believed to promote the reaction and activate the arene ring.[7-8] For arenes with multi-methoxy groups, the electron-donating groups activate the ring at the *ortho-* and *para-* positions leading to cation-radical intermediate **7**.[9-12] Overall, both types of substituents are directing and activate the arene. The methoxy group was also reported as directing group via coordination to metal catalysts,[13-16] however this mechanism was excluded since anisole (**18a**) with one methoxy group does not react (Table 1).

Table S6. Cu catalyzed chlorination using benzoyl chloride as chlorination reagent.

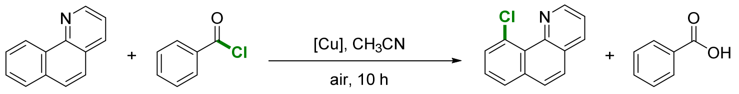

1a 4d 1c

Entry	[Cu]	**Yield (1c)**
1	$CuCl_2$	**95%**
2	$Cu(OAc)_2$	**>99%**
3	$CuSO_4$	**91%**
4	$Cu(NO_3)_2{\cdot}3H_2O$	**>99%**
5	$Cu(OAc)_2$+$NaNO_3$	**94%**
6	$NaNO_3$	**3%**

Reaction conditions: 7,8-benzoquinoline (0.3 mmol), benzoyl chloride (0.3 mmol), $Cu(NO_3)_2{\cdot}3H_2O$ (0.06 mmol), CH_3CN (2 mL), biphenyl (0.1 mmol), air (0.5 MPa), 140 ºC, 10 h.

Various Cu salts were tested and successfully promoted the chlorination reaction with benzoyl chloride as the chlorination reagent without any additives (Table S6, entries 1-4). These results demonstrated that Cu could catalyze radical-type chlorination,[7, 9] in which chlorine radicals are generated from the thermal decomposition of benzoyl chloride.[17-18]

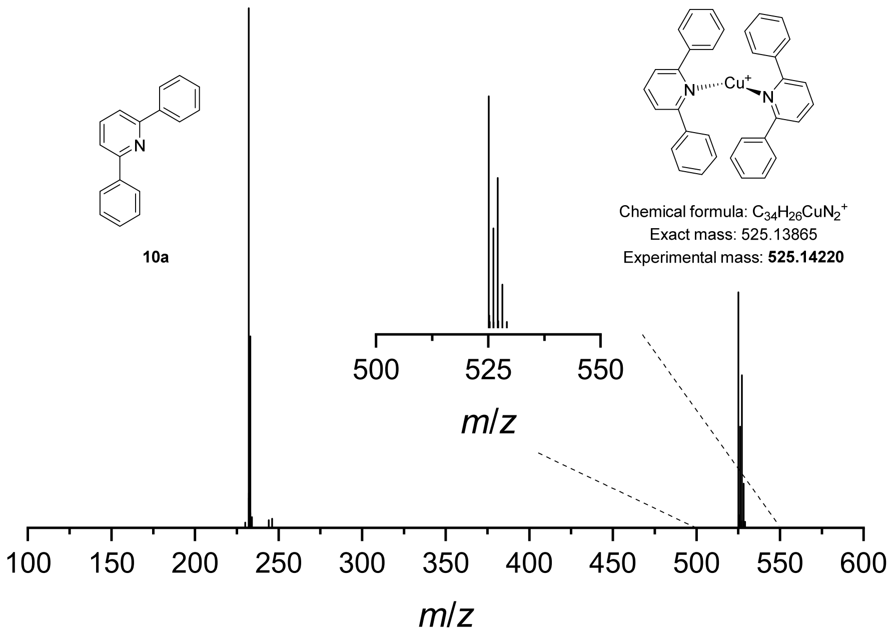

Figure S13. Electrospray ionization mass spectrum of the stable intermediate formed by the coordination of **10a** to the Cu catalyst.

After reaction, the reaction mixture was immediately diluted with CH_3CN and injected into the mass spectrometer. The unreacted substrate **10a** and a Cu complex were detected. The formation of the stable intermediate results in the low conversion of **10a**. With reactive substrates, **1a** and **2a**, stable intermediates were not detected.

NMR spectra

Substrates:

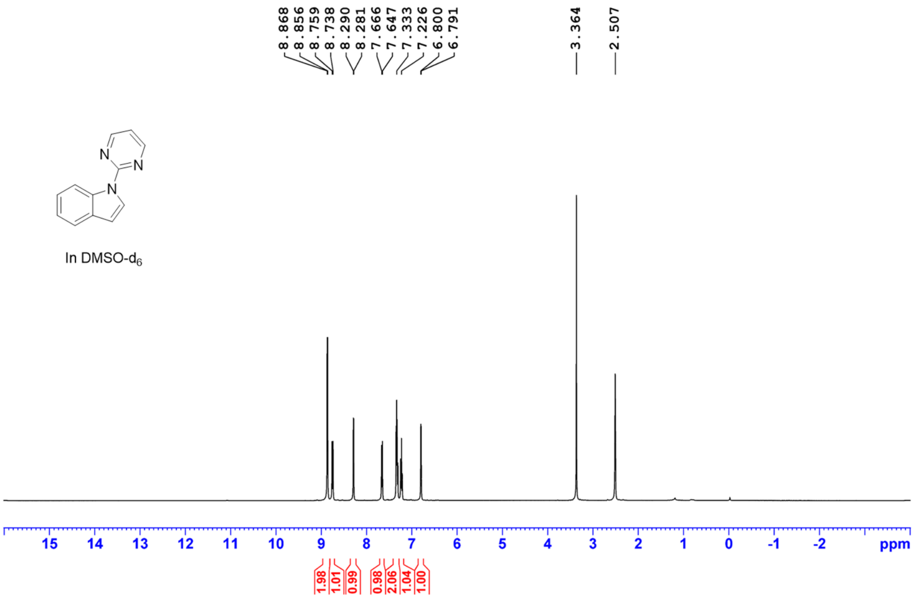

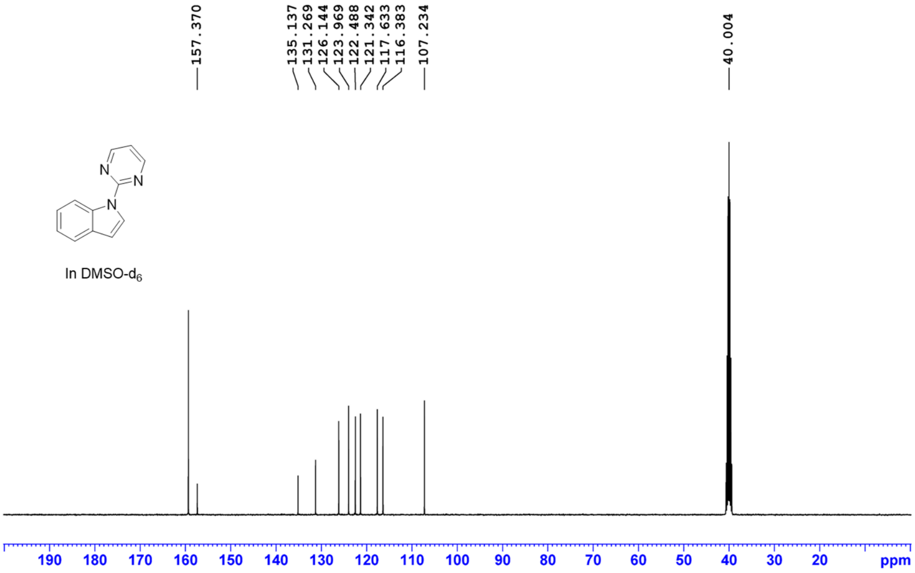

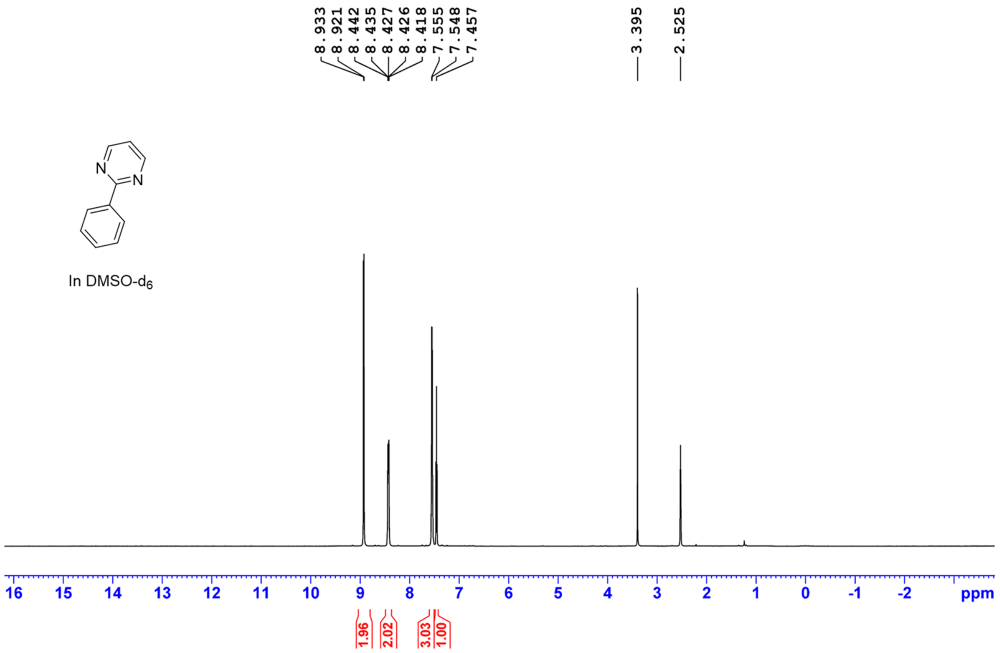

8.933
8.921
8.442
8.435
8.427
8.426
8.418
7.555
7.548
7.457
3.395
2.525
N
N
In DMSO-d6
16 15 14 13 12 11 10 9 8 7 6 5 4 3 2 1 0 -1 -2 ppm
1.96
2.02
3.03
1.00

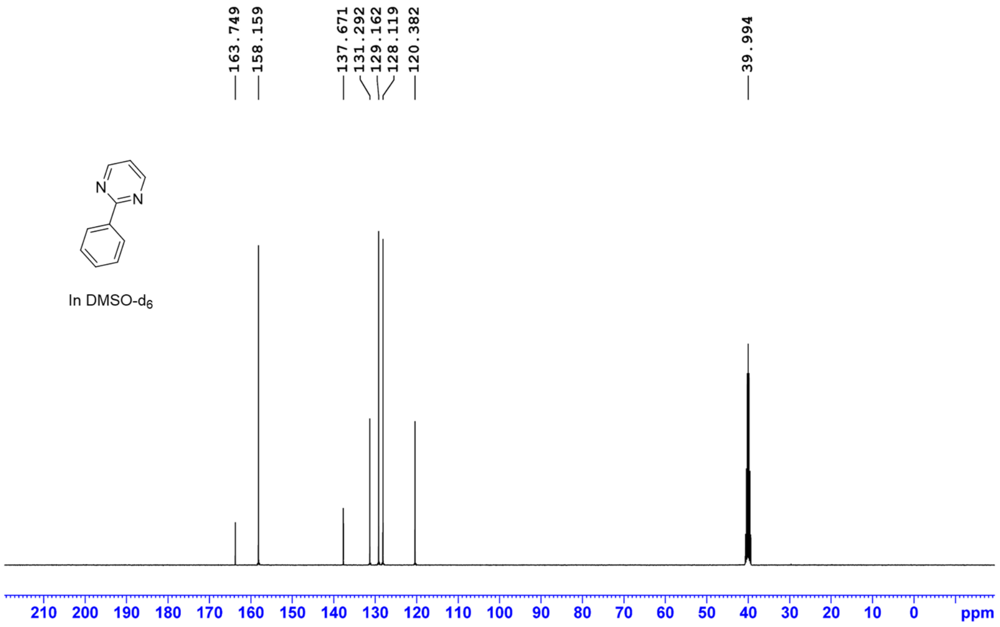

163.749
158.159
137.671
131.292
129.162
128.119
120.382
39.994
N
N
In DMSO-d6
210 200 190 180 170 160 150 140 130 120 110 100 90 80 70 60 50 40 30 20 10 0 ppm

Products:

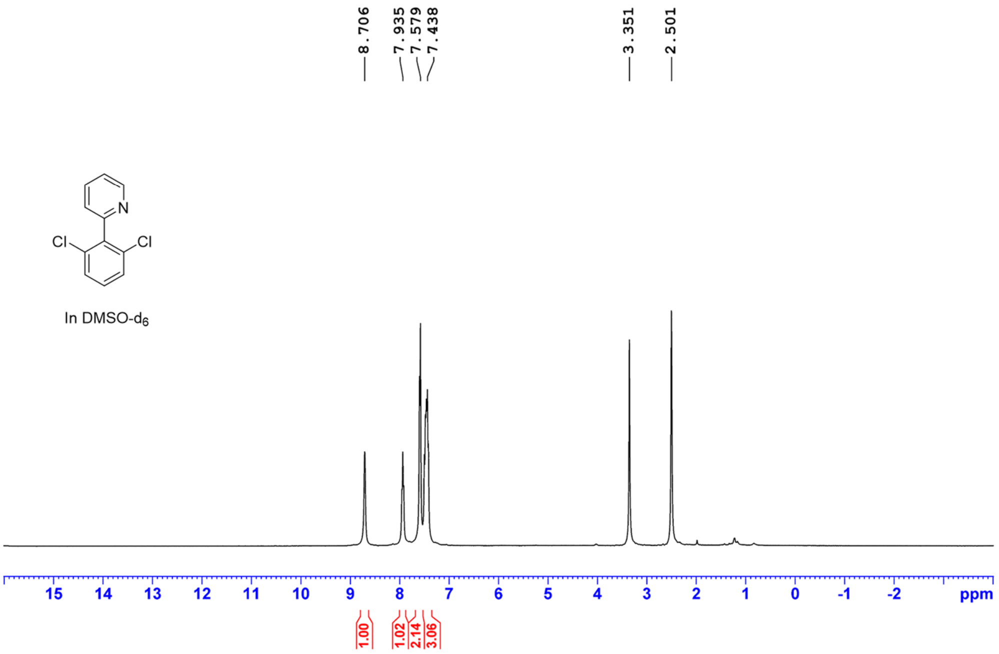
8.706
7.935
7.579
7.438
3.351
2.501
N
Cl
Cl
In DMSO-d6
15 14 13 12 11 10 9 8 7 6 5 4 3 2 1 0 -1 -2 ppm
1.00
1.02
2.14
3.06

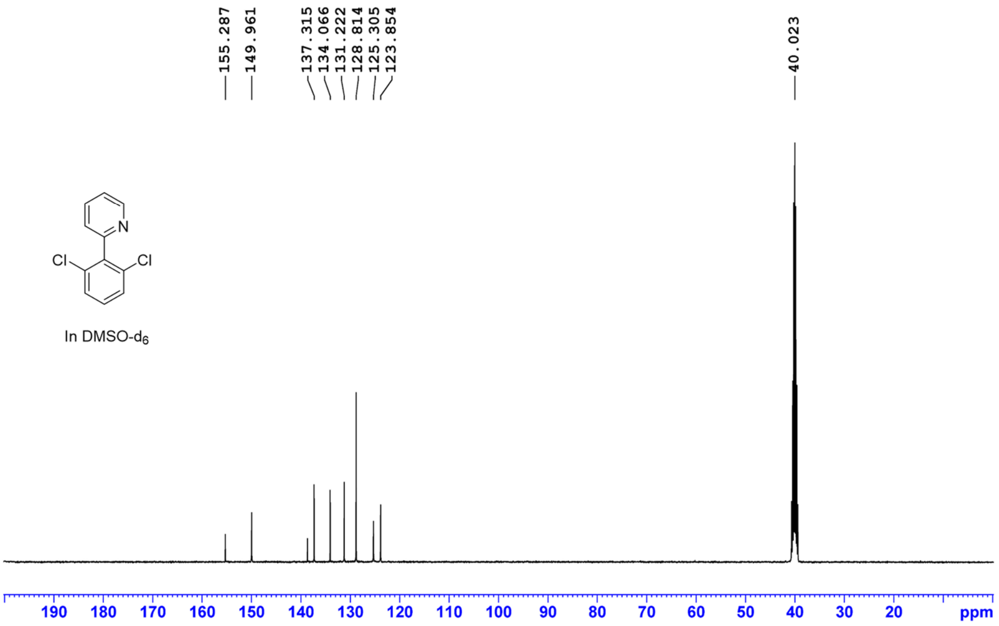
155.287
149.961
137.315
134.066
131.222
128.814
125.305
123.854
40.023
N
Cl
Cl
In DMSO-d6
190 180 170 160 150 140 130 120 110 100 90 80 70 60 50 40 30 20 ppm

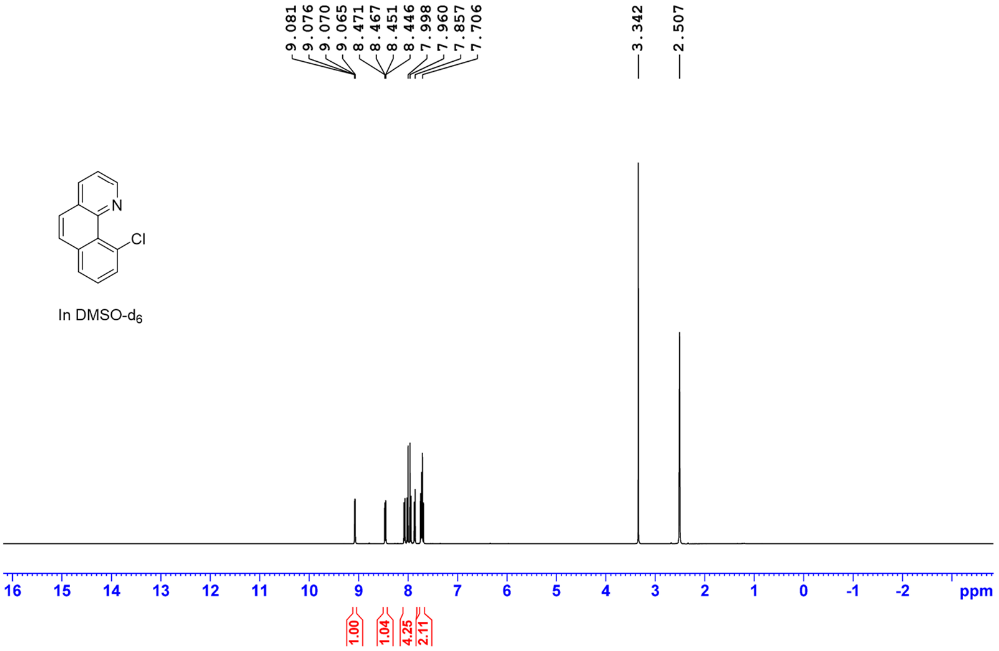
9.081
9.076
9.070
9.065
8.471
8.467
8.451
8.446
7.998
7.960
7.857
7.706
3.342
2.507
N
Cl
In DMSO-d6
16 15 14 13 12 11 10 9 8 7 6 5 4 3 2 1 0 -1 -2 ppm
1.00
1.04
4.25
2.11

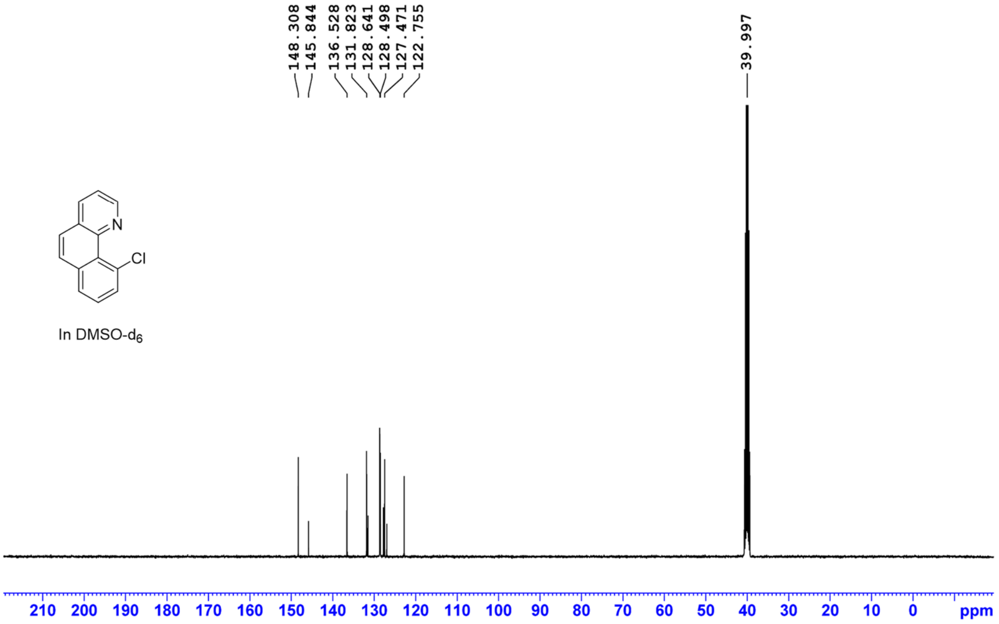
148.308
145.844
136.528
131.823
128.641
128.498
127.471
122.755
39.997
N
Cl
In DMSO-d6
210 200 190 180 170 160 150 140 130 120 110 100 90 80 70 60 50 40 30 20 10 0 ppm

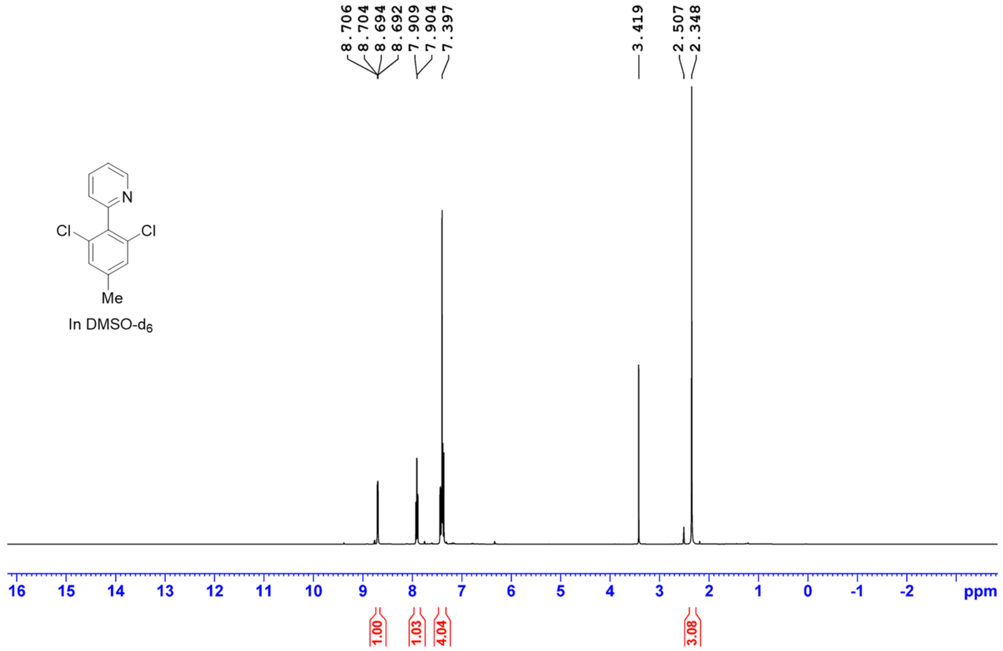
8.706
8.704
8.694
8.692
7.909
7.904
7.397
3.419
2.507
2.348
N
Cl
Cl
Me
In DMSO-d6
16 15 14 13 12 11 10 9 8 7 6 5 4 3 2 1 0 -1 -2 ppm
1.00
1.03
4.04
3.08

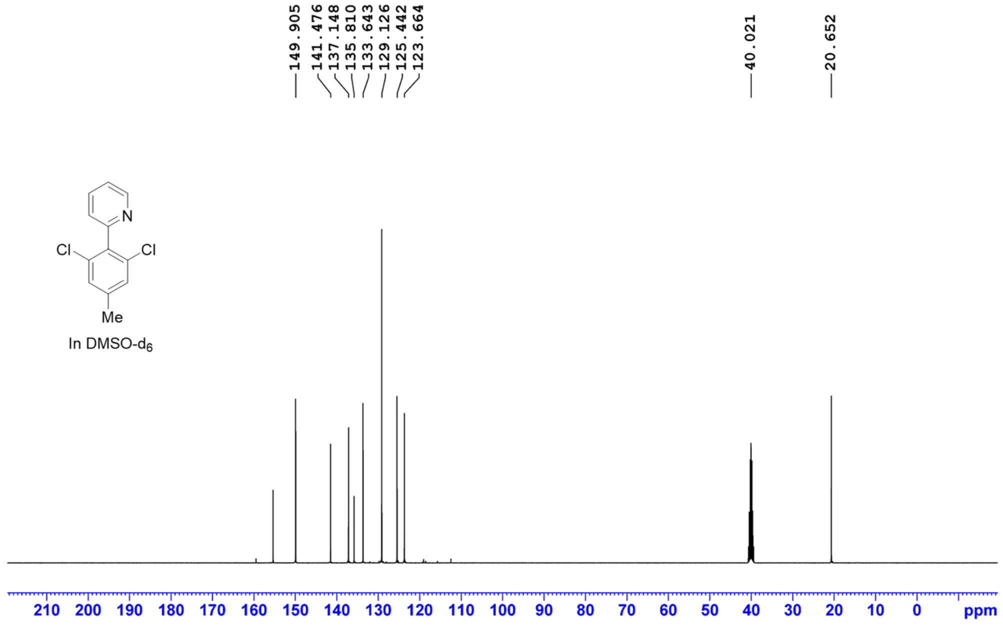
149.905
141.476
137.148
135.810
133.643
129.126
125.442
123.664
40.021
20.652
N
Cl
Cl
Me
In DMSO-d6
210 200 190 180 170 160 150 140 130 120 110 100 90 80 70 60 50 40 30 20 10 0 ppm

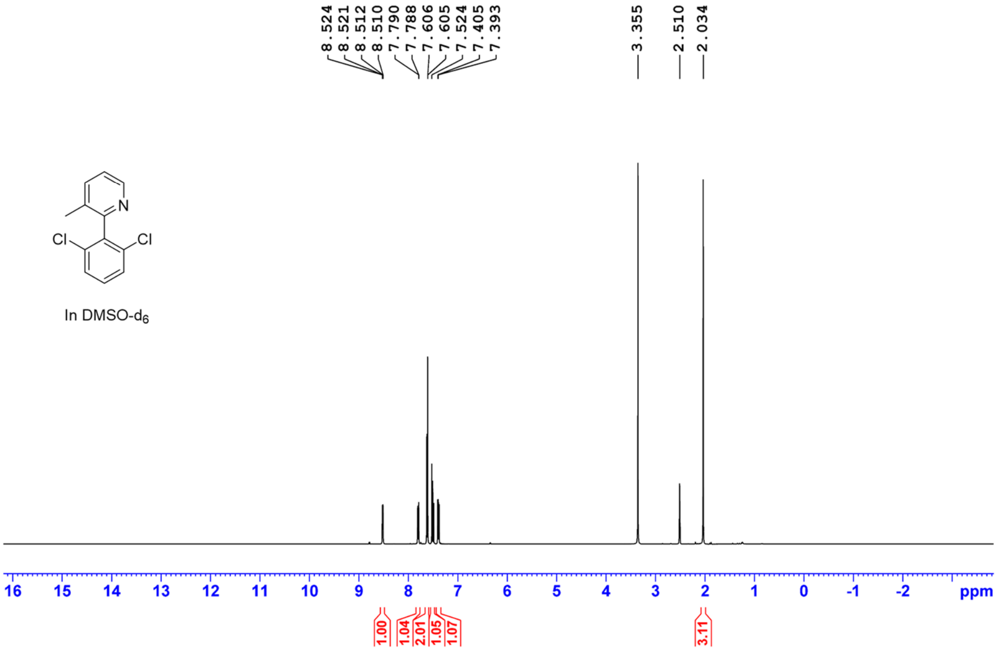
8.524
8.521
8.512
8.510
7.790
7.788
7.606
7.605
7.524
7.405
7.393
3.355
2.510
2.034
N
Cl
Cl
In DMSO-d_6
16 15 14 13 12 11 10 9 8 7 6 5 4 3 2 1 0 -1 -2 ppm
1.00
1.04
2.01
1.05
1.07
3.11

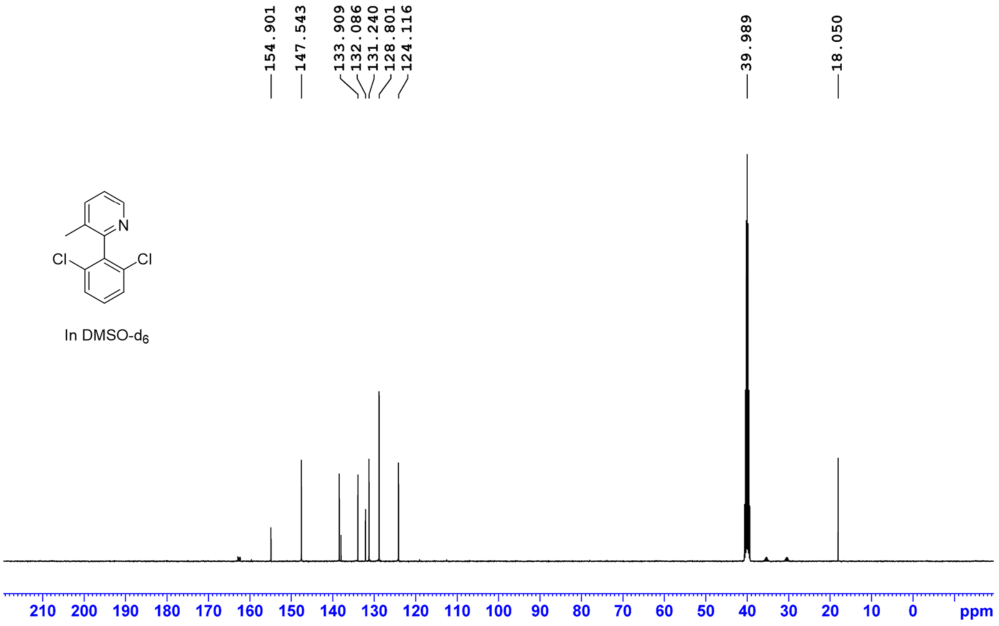
154.901
147.543
133.909
132.086
131.240
128.801
124.116
39.989
18.050
N
Cl
Cl
In DMSO-d_6
210 200 190 180 170 160 150 140 130 120 110 100 90 80 70 60 50 40 30 20 10 0 ppm

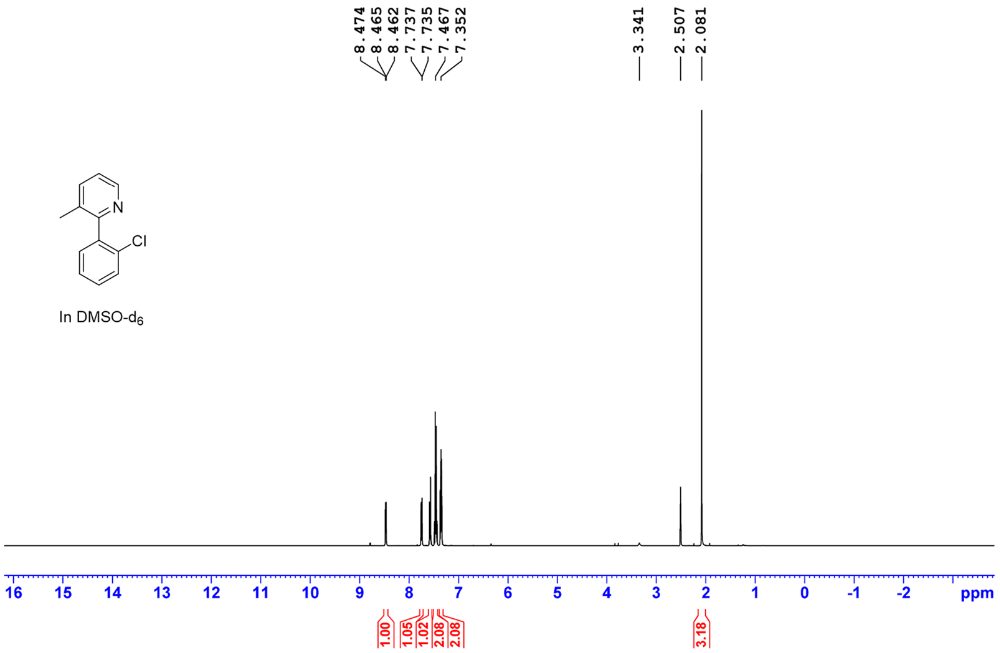
8.474
8.465
8.462
7.737
7.735
7.467
7.352
3.341
2.507
2.081
N
Cl
In DMSO-d6
16 15 14 13 12 11 10 9 8 7 6 5 4 3 2 1 0 -1 -2 ppm
1.00
1.05
1.02
2.08
2.08
3.18

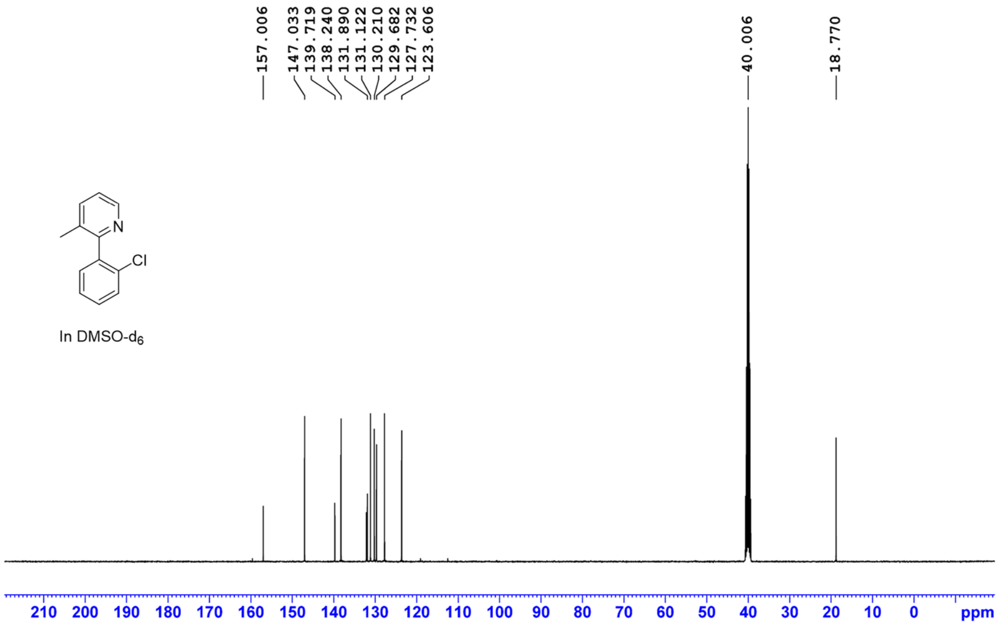
157.006
147.033
139.719
138.240
131.890
131.122
130.210
129.682
127.732
123.606
40.006
18.770
N
Cl
In DMSO-d6
210 200 190 180 170 160 150 140 130 120 110 100 90 80 70 60 50 40 30 20 10 0 ppm

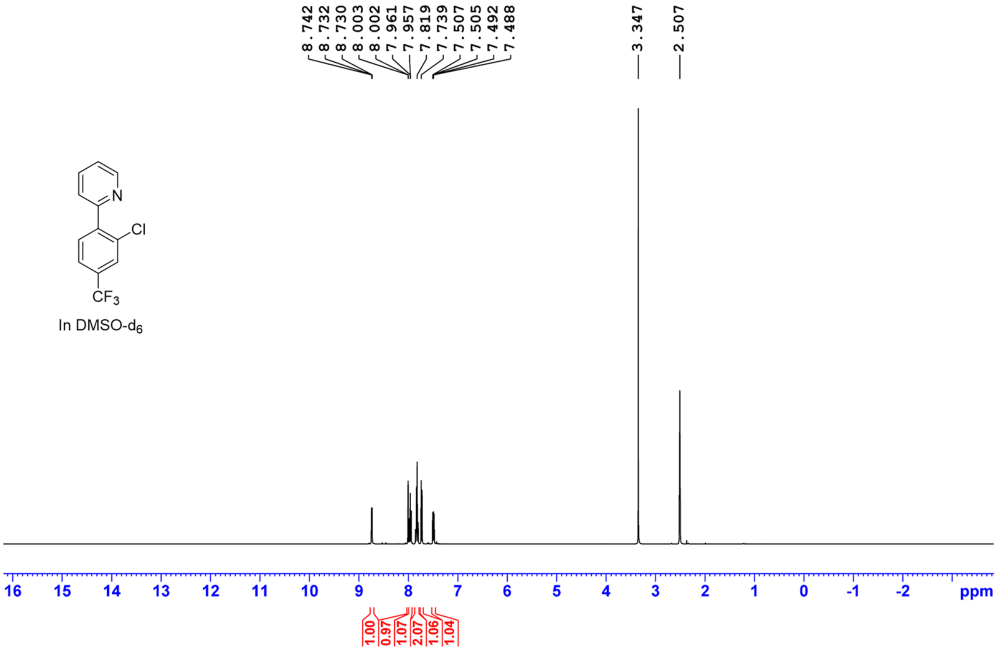
8.742
8.732
8.730
8.003
8.002
7.961
7.957
7.819
7.739
7.507
7.505
7.492
7.488
3.347
2.507
In DMSO-d6
1.00
0.97
1.07
2.07
1.06
1.04
ppm

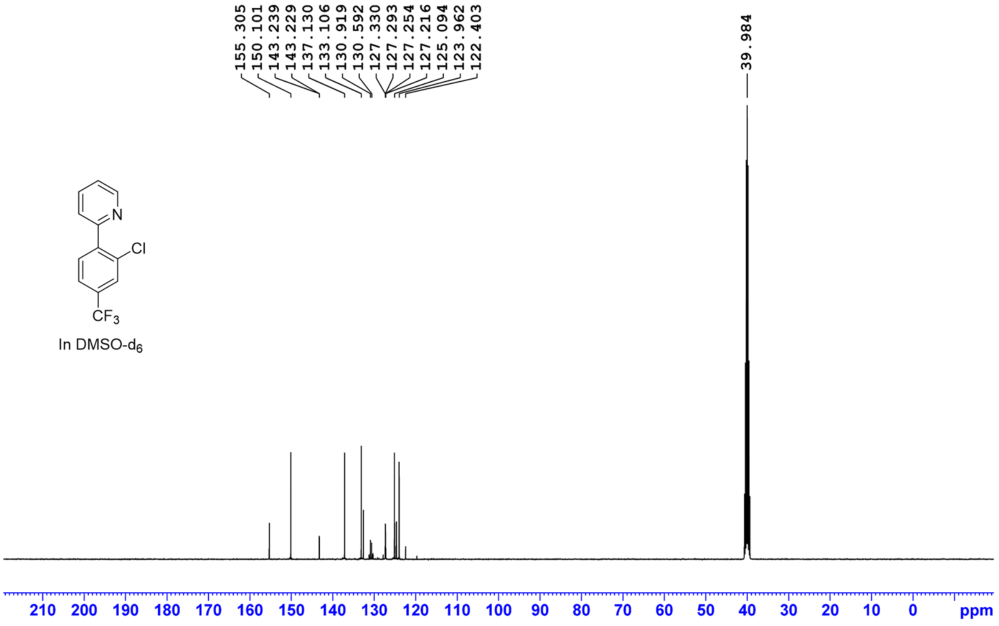
155.305
150.101
143.239
143.229
137.130
133.106
130.919
130.592
127.330
127.293
127.254
127.216
125.094
123.962
122.403
39.984
In DMSO-d6
ppm

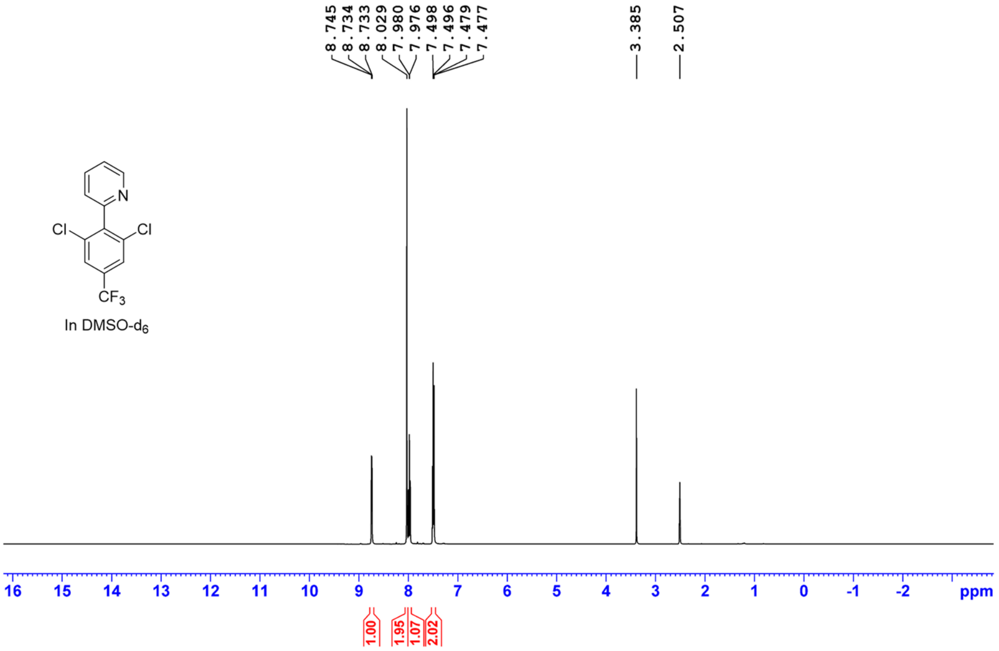
8.745
8.734
8.733
8.029
7.980
7.976
7.498
7.496
7.479
7.477
3.385
2.507
Cl
Cl
N
CF3
In DMSO-d6
16 15 14 13 12 11 10 9 8 7 6 5 4 3 2 1 0 -1 -2 ppm
1.00
1.95
1.07
2.02

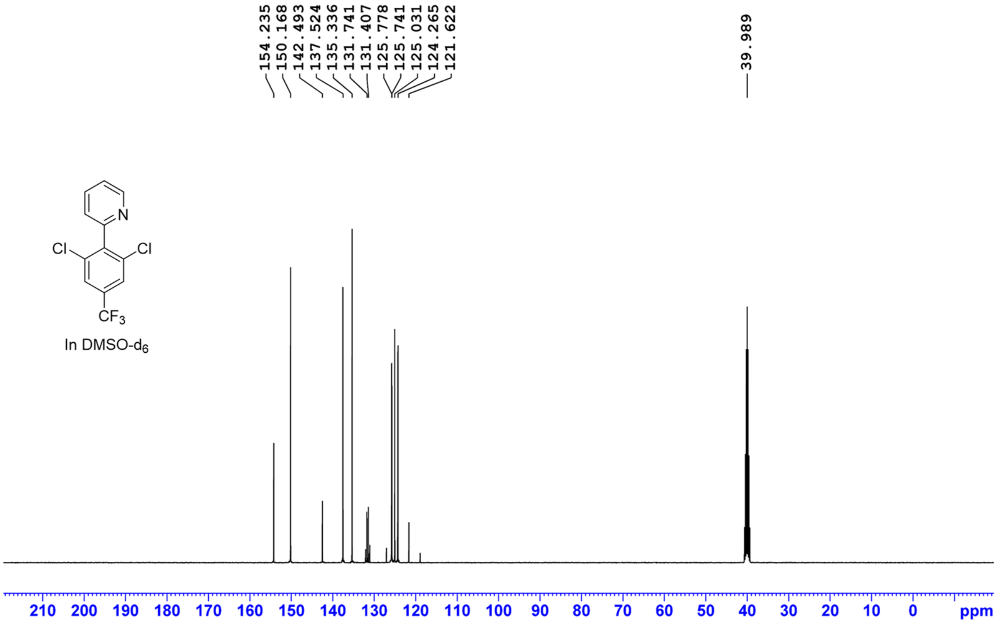
154.235
150.168
142.493
137.524
135.336
131.741
131.407
125.778
125.741
125.031
124.265
121.622
39.989
Cl
Cl
N
CF3
In DMSO-d6
210 200 190 180 170 160 150 140 130 120 110 100 90 80 70 60 50 40 30 20 10 0 ppm

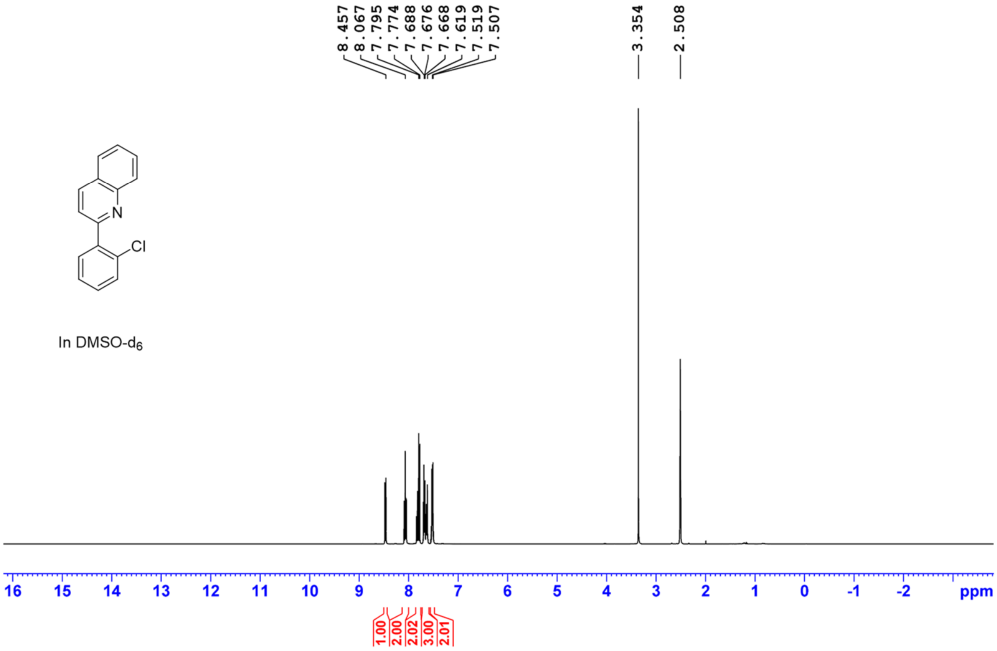

8.457
8.067
7.795
7.774
7.688
7.676
7.668
7.619
7.519
7.507
3.354
2.508
N
Cl
In DMSO-d6
16 15 14 13 12 11 10 9 8 7 6 5 4 3 2 1 0 -1 -2 ppm
1.00
2.00
2.02
3.00
2.01

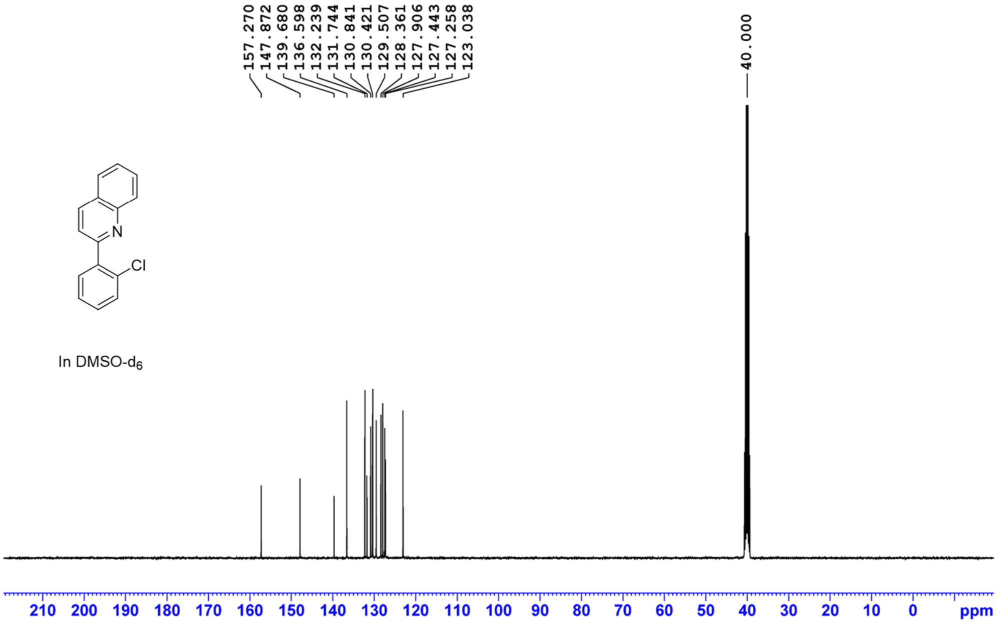

157.270
147.872
139.680
136.598
132.239
131.744
130.841
130.421
129.507
128.361
127.906
127.443
127.258
123.038
40.000
N
Cl
In DMSO-d6
210 200 190 180 170 160 150 140 130 120 110 100 90 80 70 60 50 40 30 20 10 0 ppm

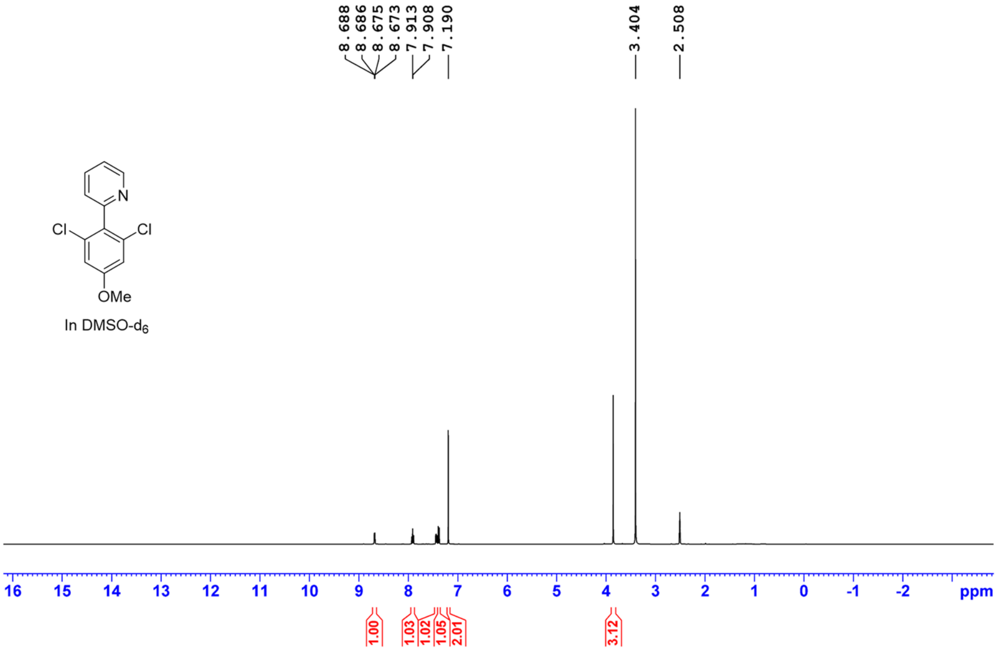
8.688
8.686
8.675
8.673
7.913
7.908
7.190
3.404
2.508
In DMSO-d6
1.00
1.03
1.02
1.05
2.01
3.12
ppm

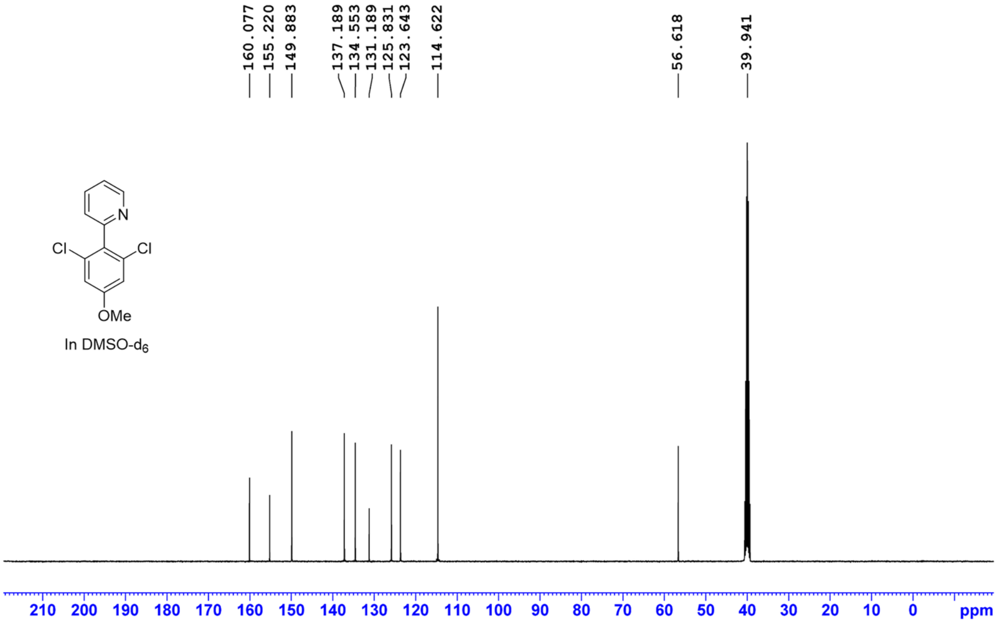
160.077
155.220
149.883
137.189
134.553
131.189
125.831
123.643
114.622
56.618
39.941
In DMSO-d6
ppm

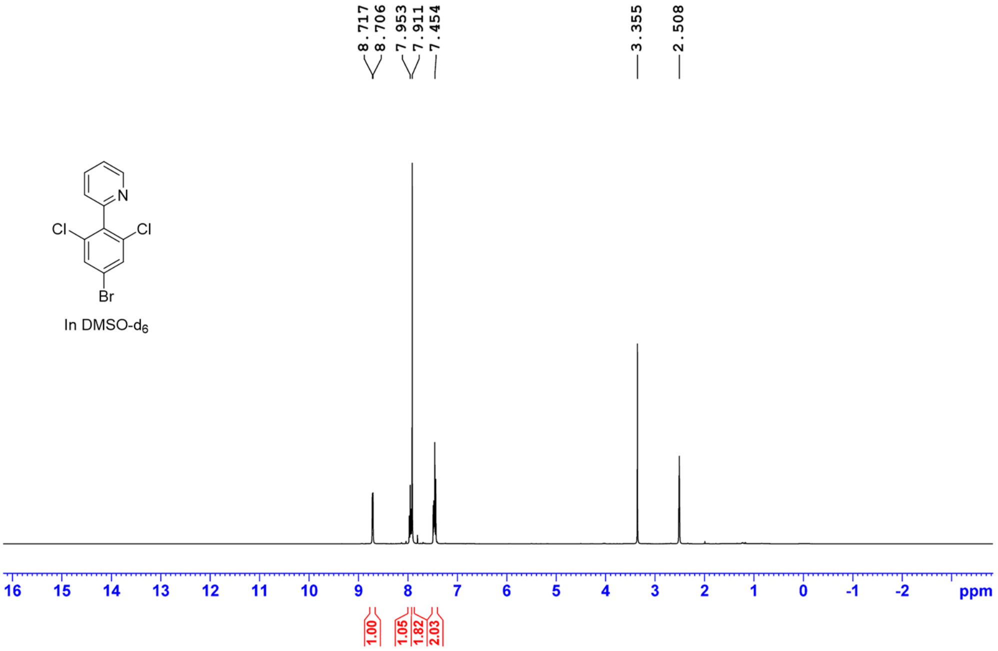
8.717
8.706
7.953
7.911
7.454
3.355
2.508
N
Cl
Cl
Br
In DMSO-d6
16 15 14 13 12 11 10 9 8 7 6 5 4 3 2 1 0 -1 -2 ppm
1.00
1.05
1.82
2.03

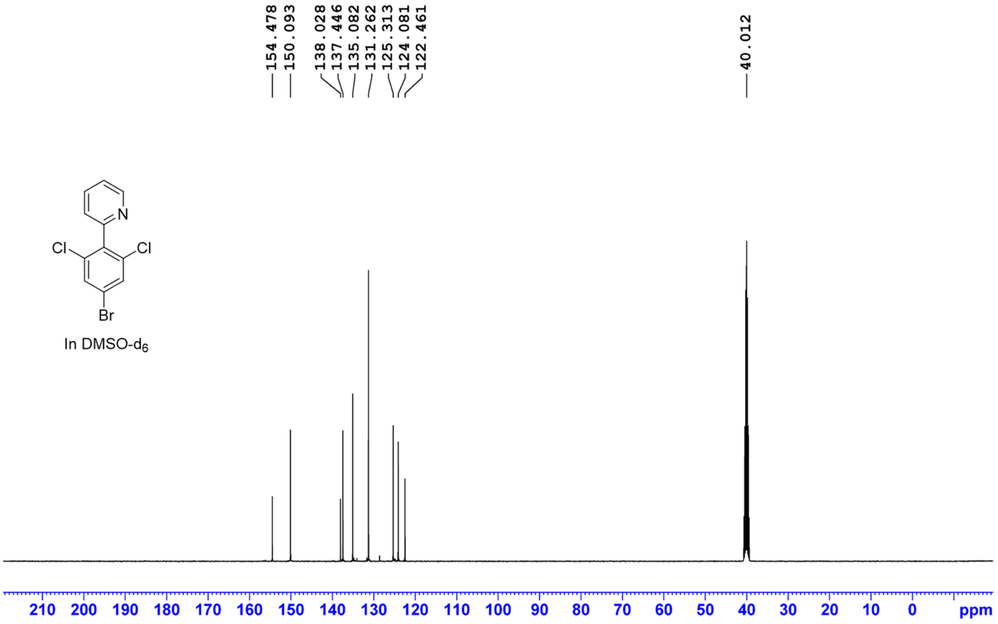
154.478
150.093
138.028
137.446
135.082
131.262
125.313
124.081
122.461
40.012
N
Cl
Cl
Br
In DMSO-d6
210 200 190 180 170 160 150 140 130 120 110 100 90 80 70 60 50 40 30 20 10 0 ppm

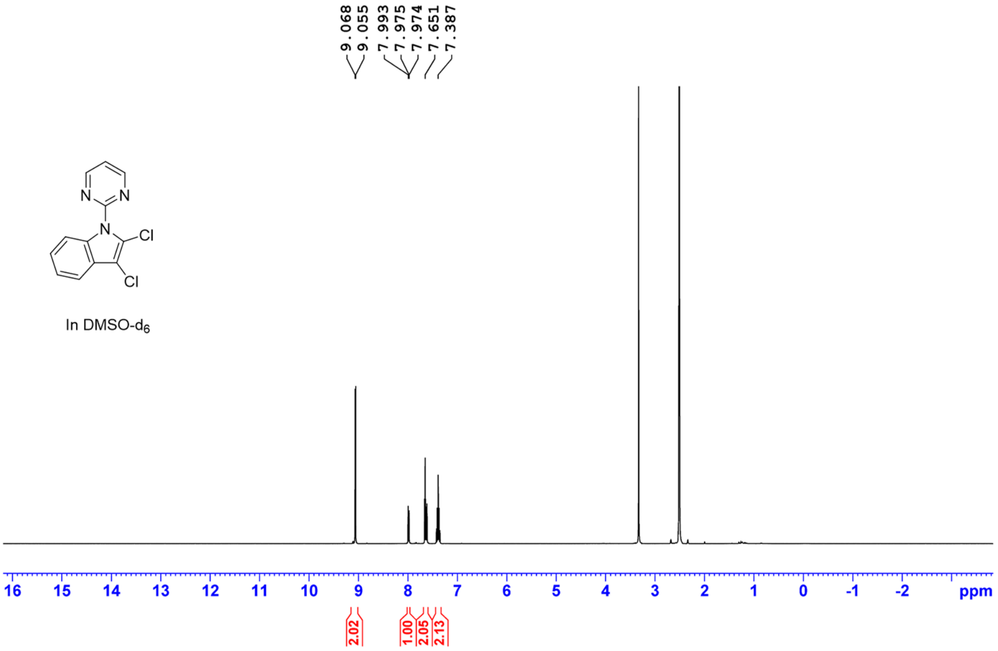

9.068
9.055
7.993
7.975
7.974
7.651
7.387
N
N
N
Cl
Cl
In DMSO-d6
16 15 14 13 12 11 10 9 8 7 6 5 4 3 2 1 0 -1 -2 ppm
2.02
1.00
2.05
2.13

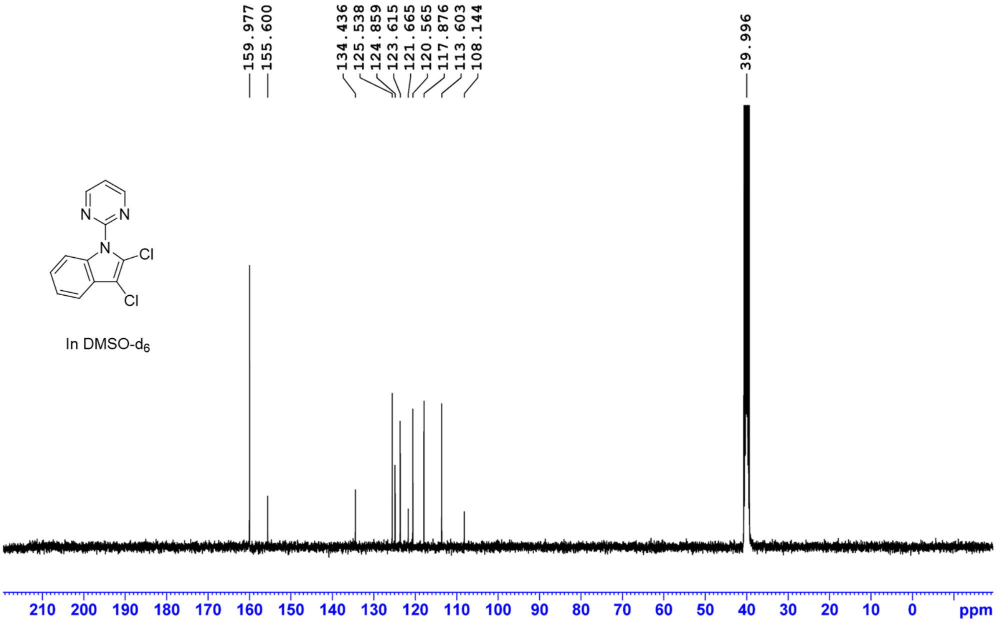

159.977
155.600
134.436
125.538
124.859
123.615
121.665
120.565
117.876
113.603
108.144
39.996
N
N
N
Cl
Cl
In DMSO-d6
210 200 190 180 170 160 150 140 130 120 110 100 90 80 70 60 50 40 30 20 10 0 ppm

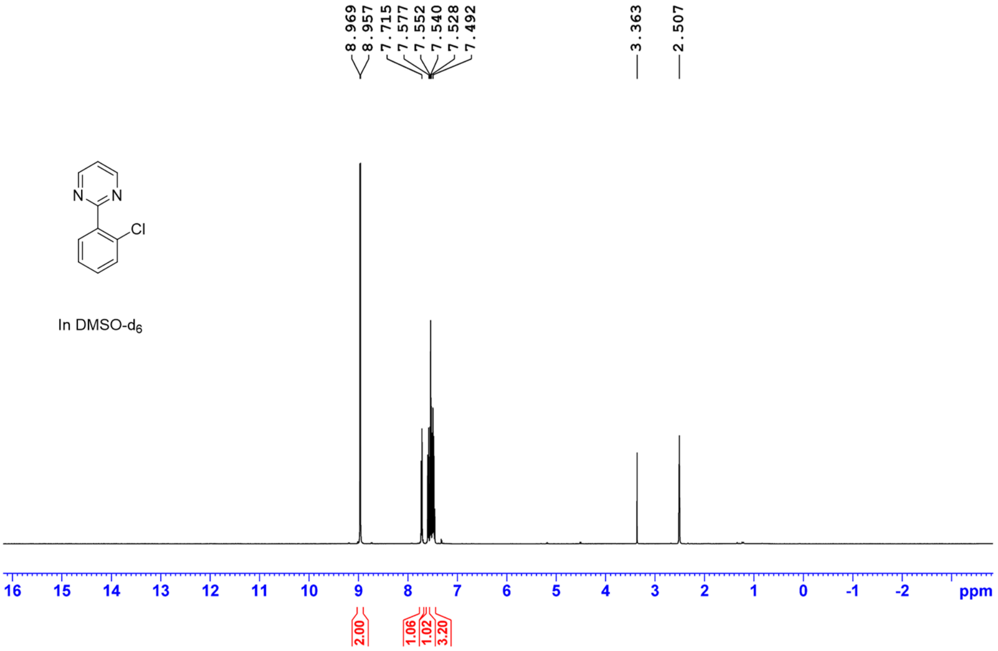
8.969
8.957
7.715
7.577
7.552
7.540
7.528
7.492
3.363
2.507
N
N
Cl
In DMSO-d6
16 15 14 13 12 11 10 9 8 7 6 5 4 3 2 1 0 -1 -2 ppm
2.00
1.06
1.02
3.20

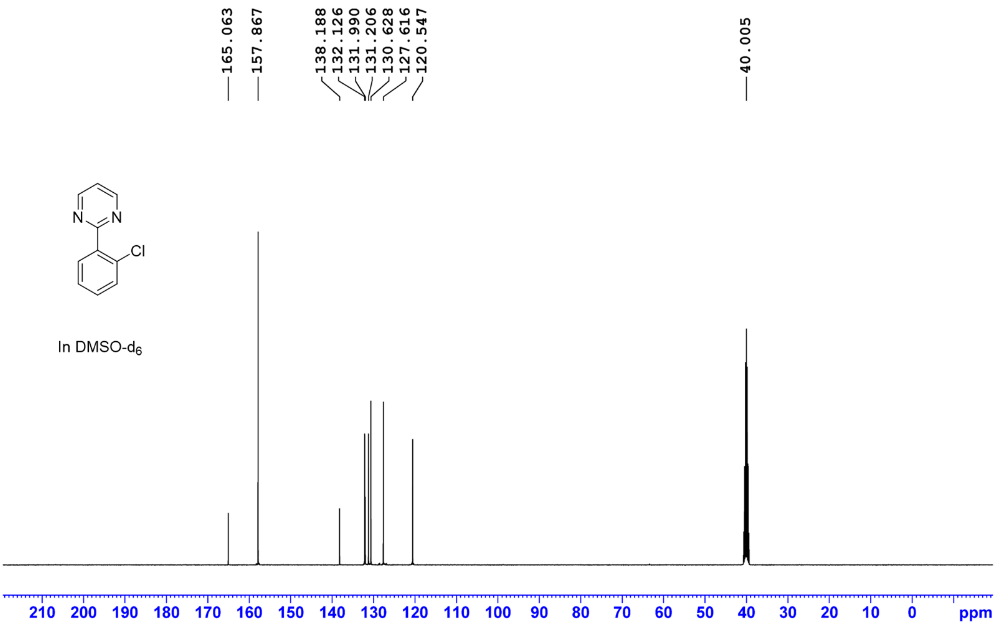
165.063
157.867
138.188
132.126
131.990
131.206
130.628
127.616
120.547
40.005
N
N
Cl
In DMSO-d6
210 200 190 180 170 160 150 140 130 120 110 100 90 80 70 60 50 40 30 20 10 0 ppm

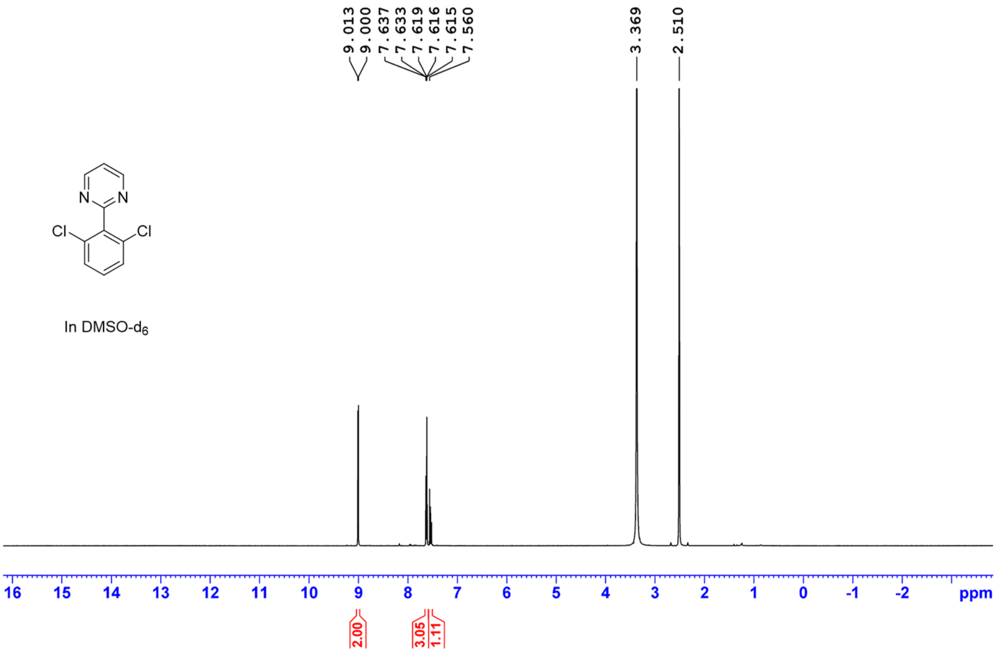
9.013
9.000
7.637
7.633
7.619
7.616
7.615
7.560
3.369
2.510
N
N
Cl
Cl
In DMSO-d6
16 15 14 13 12 11 10 9 8 7 6 5 4 3 2 1 0 -1 -2 ppm
2.00
3.05
1.11

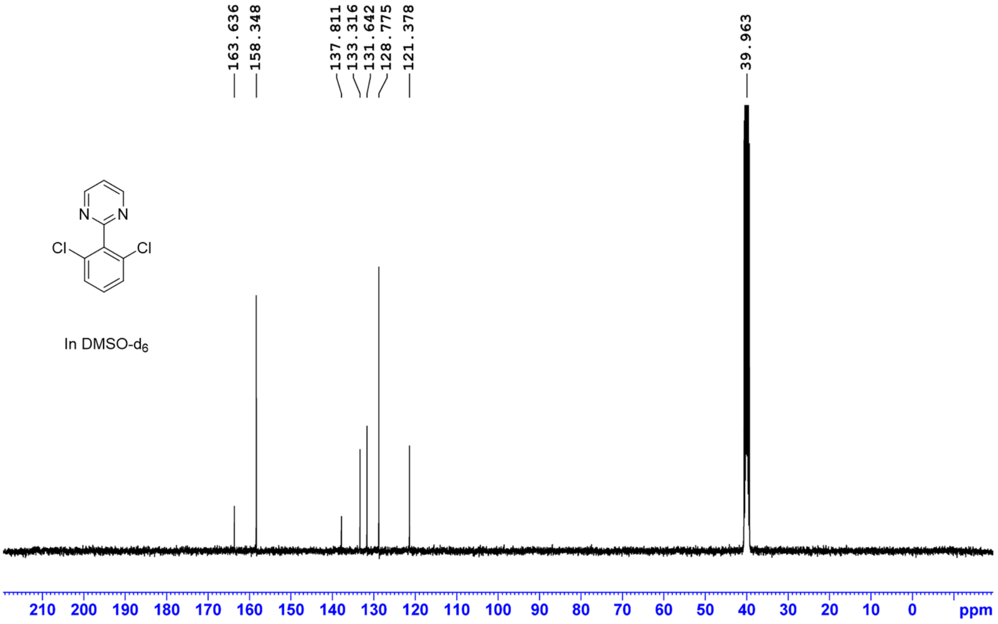
163.636
158.348
137.811
133.316
131.642
128.775
121.378
39.963
N
N
Cl
Cl
In DMSO-d6
210 200 190 180 170 160 150 140 130 120 110 100 90 80 70 60 50 40 30 20 10 0 ppm

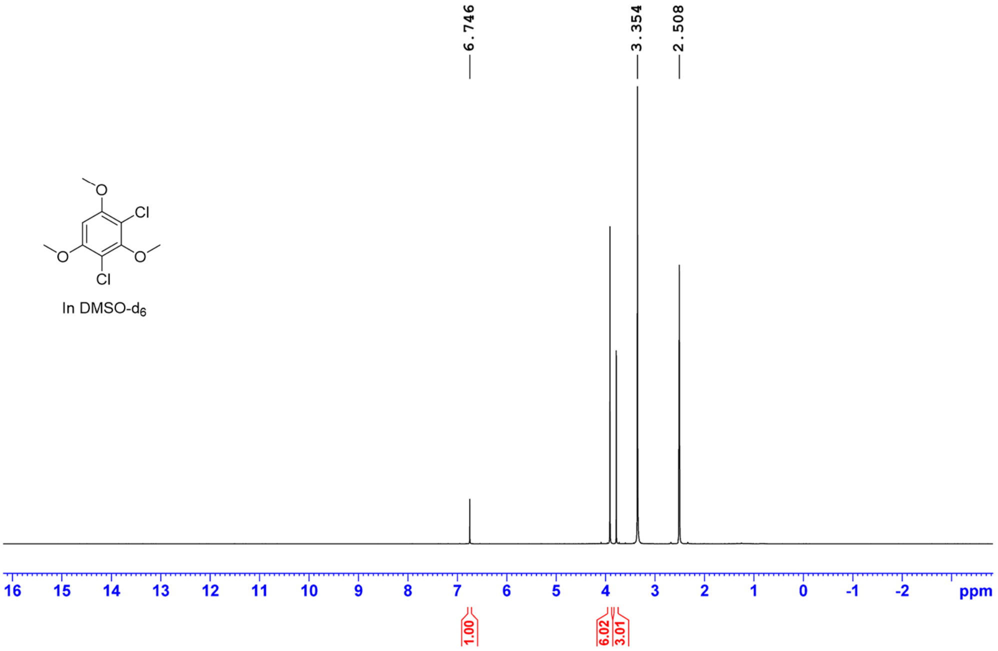

6.746
3.354
2.508
Cl
O
O
O
Cl
In DMSO-d6
16
15
14
13
12
11
10
9
8
7
6
5
4
3
2
1
0
-1
-2
ppm
1.00
6.02
3.01

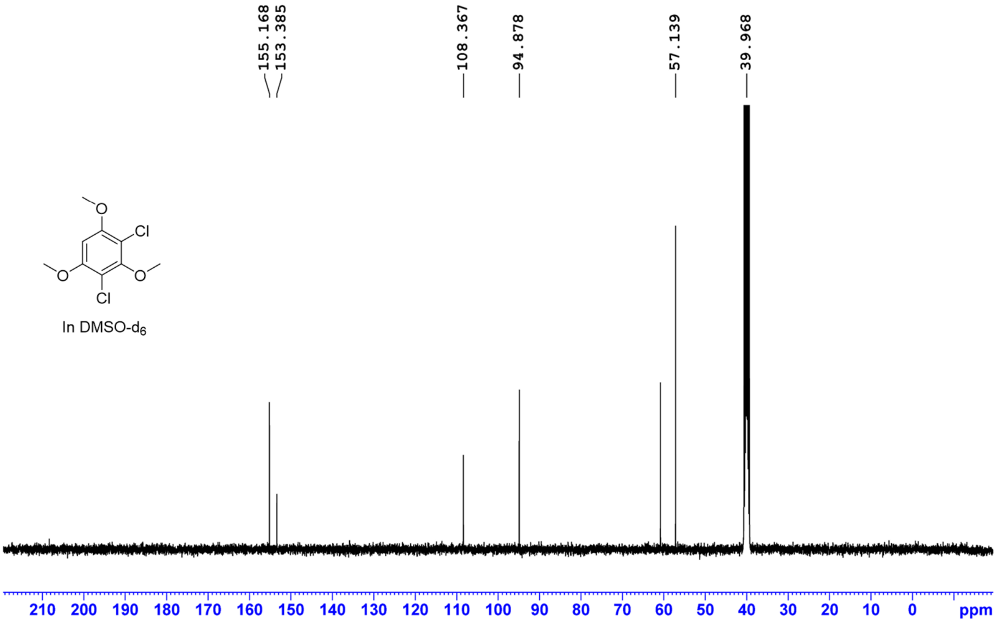

155.168
153.385
108.367
94.878
57.139
39.968
Cl
O
O
O
Cl
In DMSO-d6
210
200
190
180
170
160
150
140
130
120
110
100
90
80
70
60
50
40
30
20
10
0
ppm

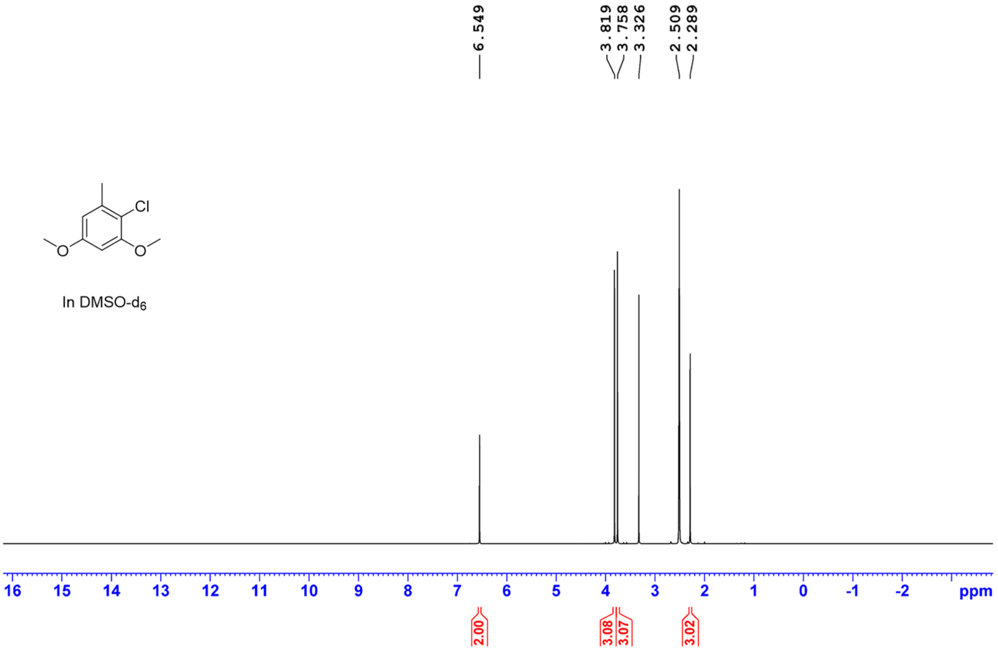
6.549
3.819
3.758
3.326
2.509
2.289
Cl
O
O
In DMSO-d6
16 15 14 13 12 11 10 9 8 7 6 5 4 3 2 1 0 -1 -2 ppm
2.00
3.08
3.07
3.02

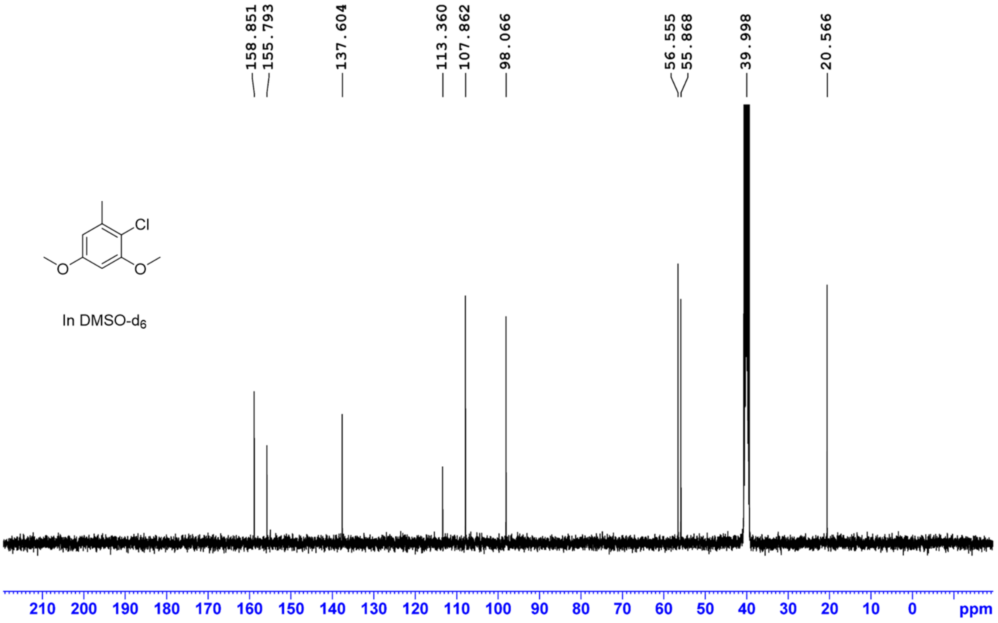
158.851
155.793
137.604
113.360
107.862
98.066
56.555
55.868
39.998
20.566
Cl
O
O
In DMSO-d6
210 200 190 180 170 160 150 140 130 120 110 100 90 80 70 60 50 40 30 20 10 0 ppm

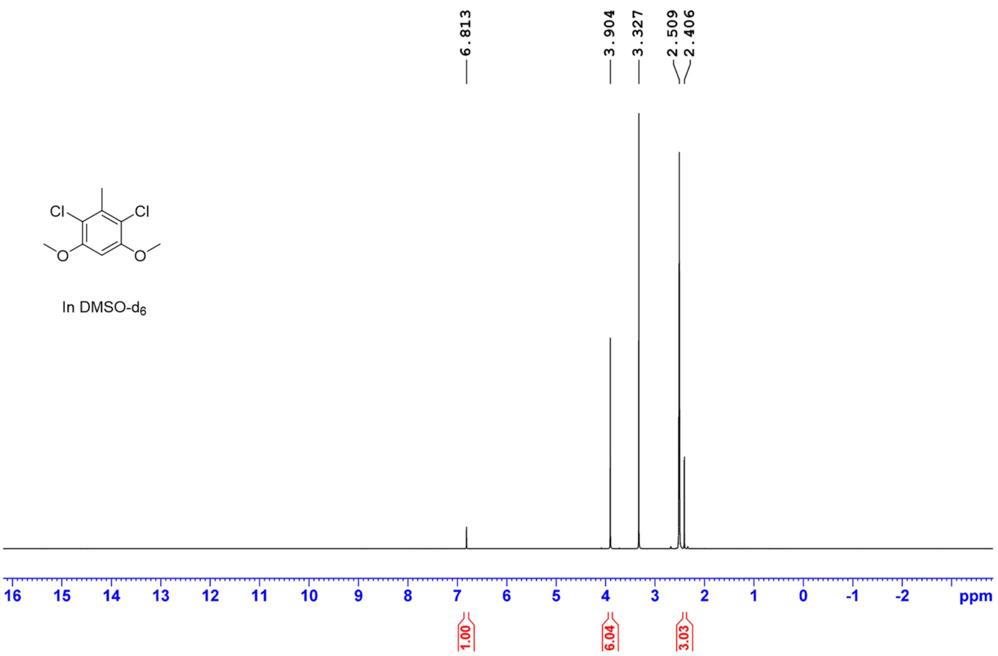
6.813
3.904
3.327
2.509
2.406
Cl
Cl
O
O
In DMSO-d_6
16 15 14 13 12 11 10 9 8 7 6 5 4 3 2 1 0 -1 -2 ppm
1.00
6.04
3.03

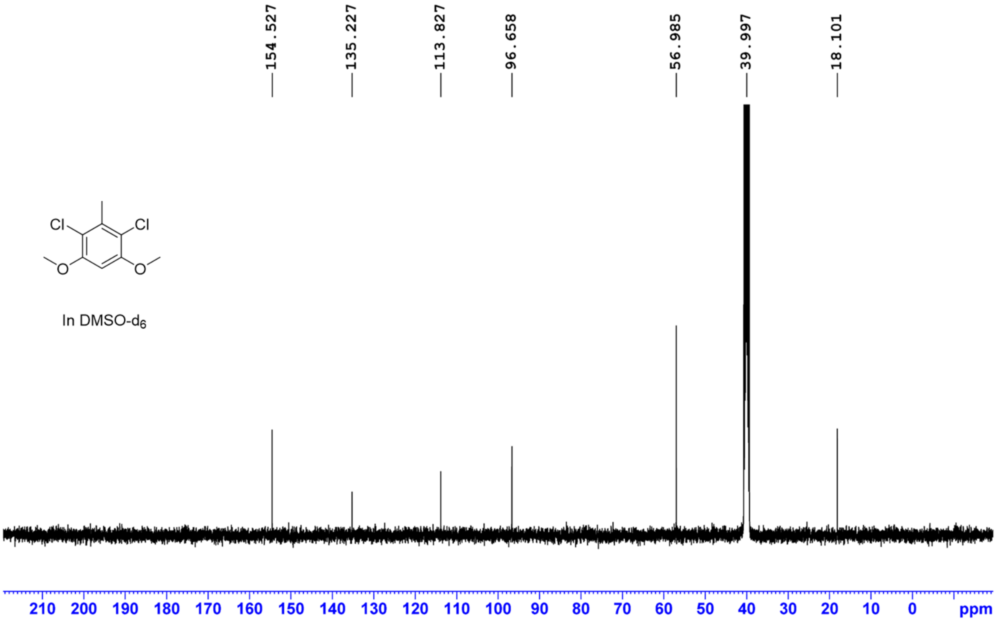
154.527
135.227
113.827
96.658
56.985
39.997
18.101
Cl
Cl
O
O
In DMSO-d_6
210 200 190 180 170 160 150 140 130 120 110 100 90 80 70 60 50 40 30 20 10 0 ppm

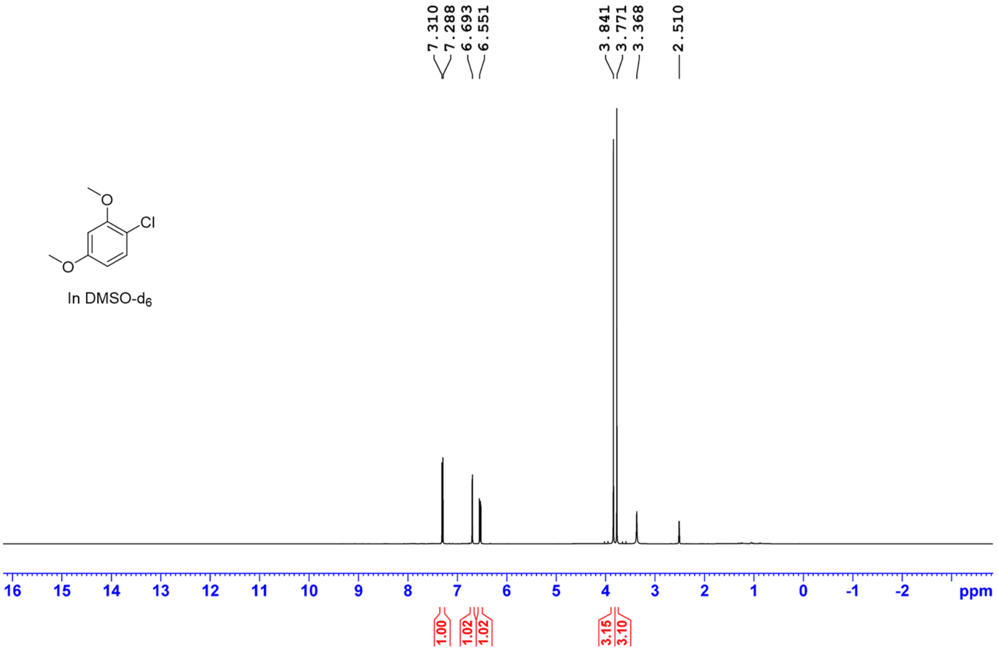
7.310
7.288
6.693
6.551
3.841
3.771
3.368
2.510
Cl
O
O
In DMSO-d6
16 15 14 13 12 11 10 9 8 7 6 5 4 3 2 1 0 -1 -2 ppm
1.00
1.02
1.02
3.15
3.10

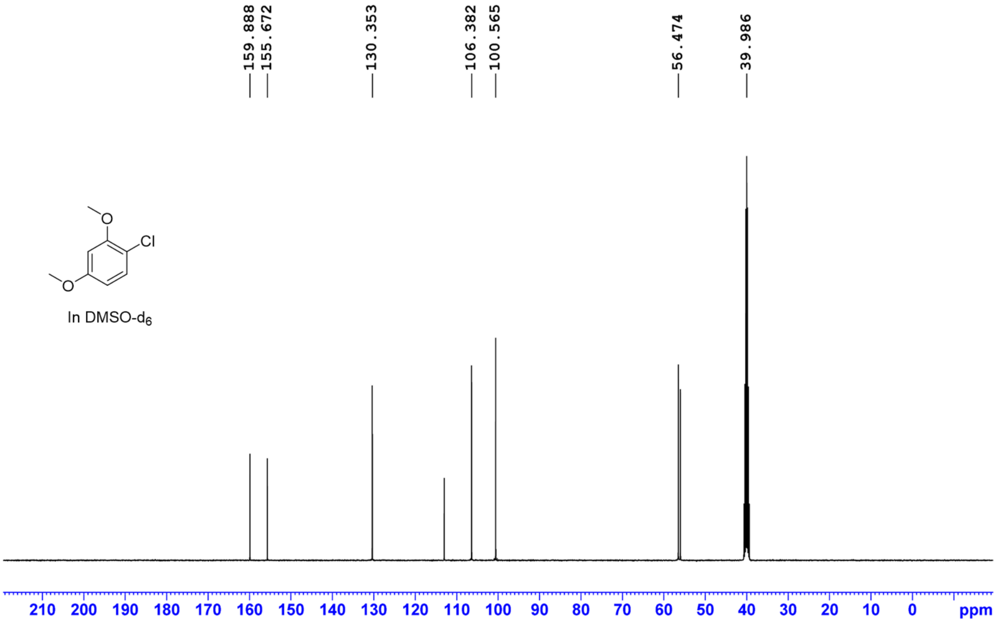
159.888
155.672
130.353
106.382
100.565
56.474
39.986
Cl
O
O
In DMSO-d6
210 200 190 180 170 160 150 140 130 120 110 100 90 80 70 60 50 40 30 20 10 0 ppm

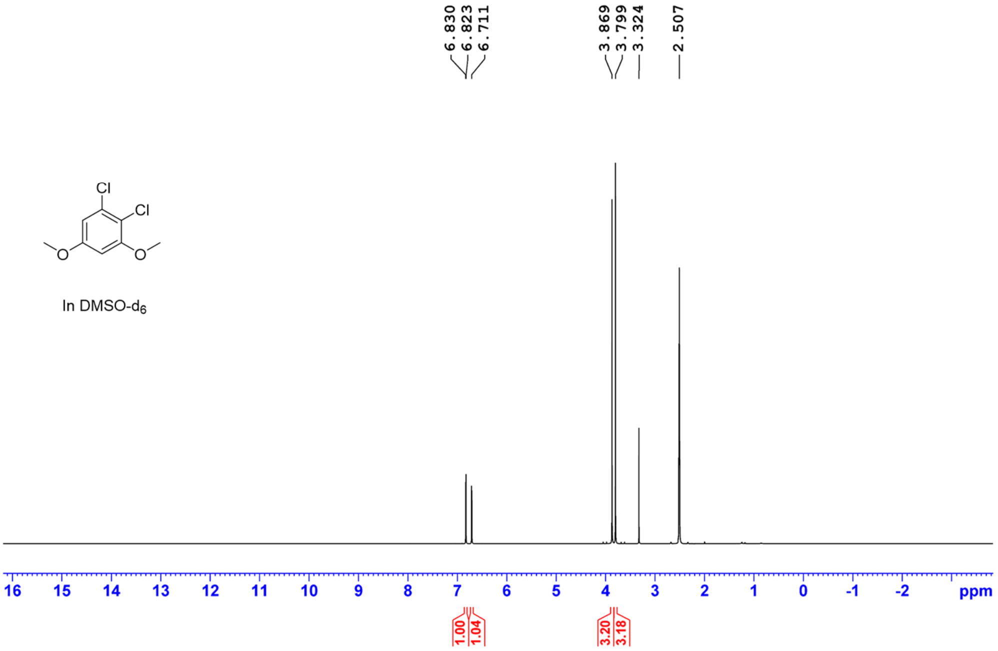
6.830
6.823
6.711
3.869
3.799
3.324
2.507
Cl
Cl
O
O
In DMSO-d6
1.00
1.04
3.20
3.18
ppm

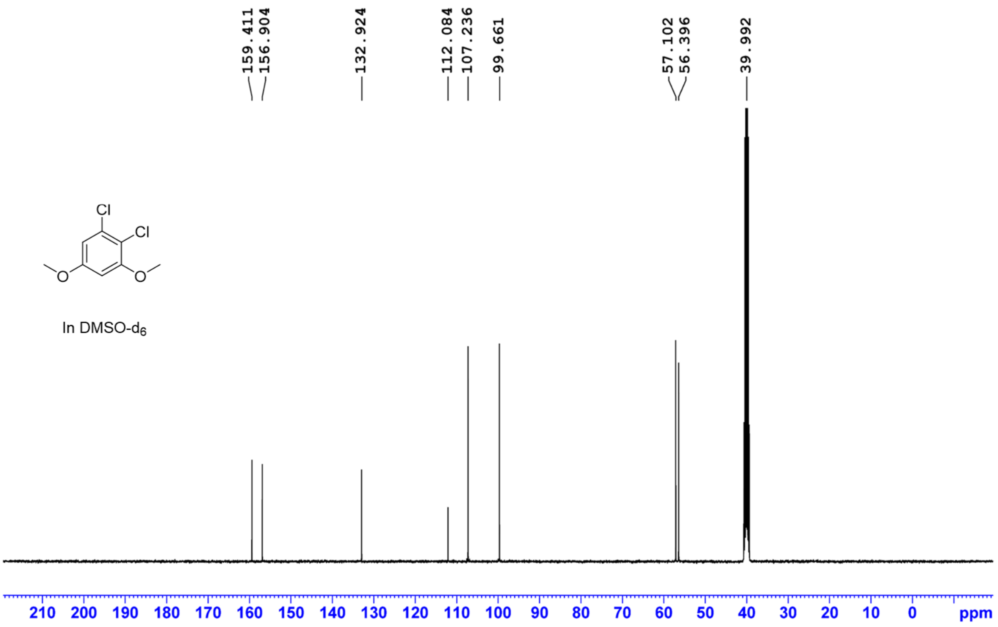
159.411
156.904
132.924
112.084
107.236
99.661
57.102
56.396
39.992
Cl
Cl
O
O
In DMSO-d6
ppm

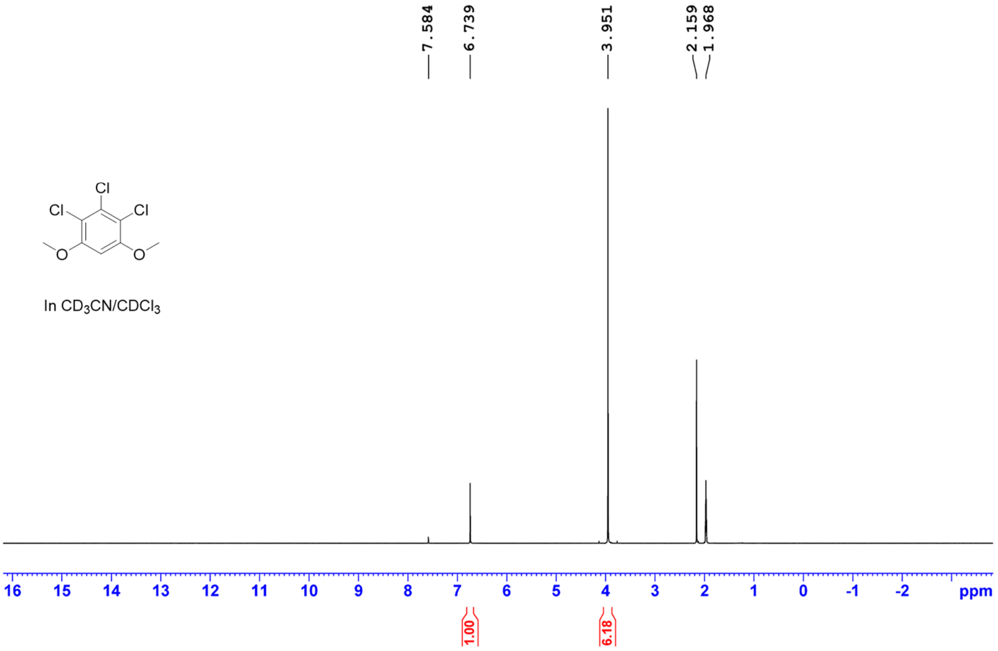
7.584
6.739
3.951
2.159
1.968
Cl
Cl
Cl
O
O
In $CD_3CN/CDCl_3$
16 15 14 13 12 11 10 9 8 7 6 5 4 3 2 1 0 -1 -2 ppm
1.00
6.18

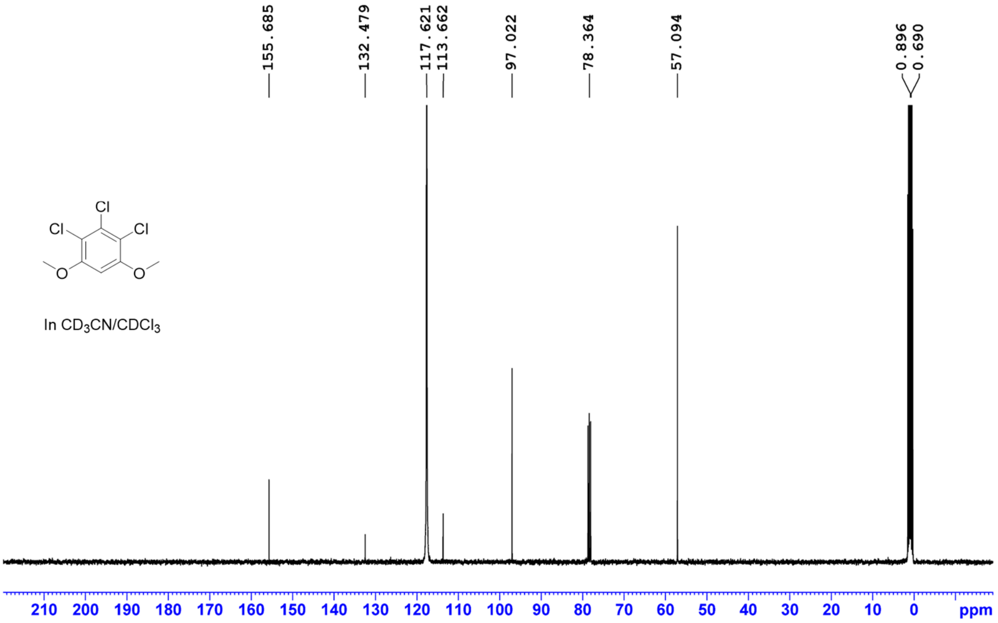
155.685
132.479
117.621
113.662
97.022
78.364
57.094
0.896
0.690
Cl
Cl
Cl
O
O
In $CD_3CN/CDCl_3$
210 200 190 180 170 160 150 140 130 120 110 100 90 80 70 60 50 40 30 20 10 0 ppm

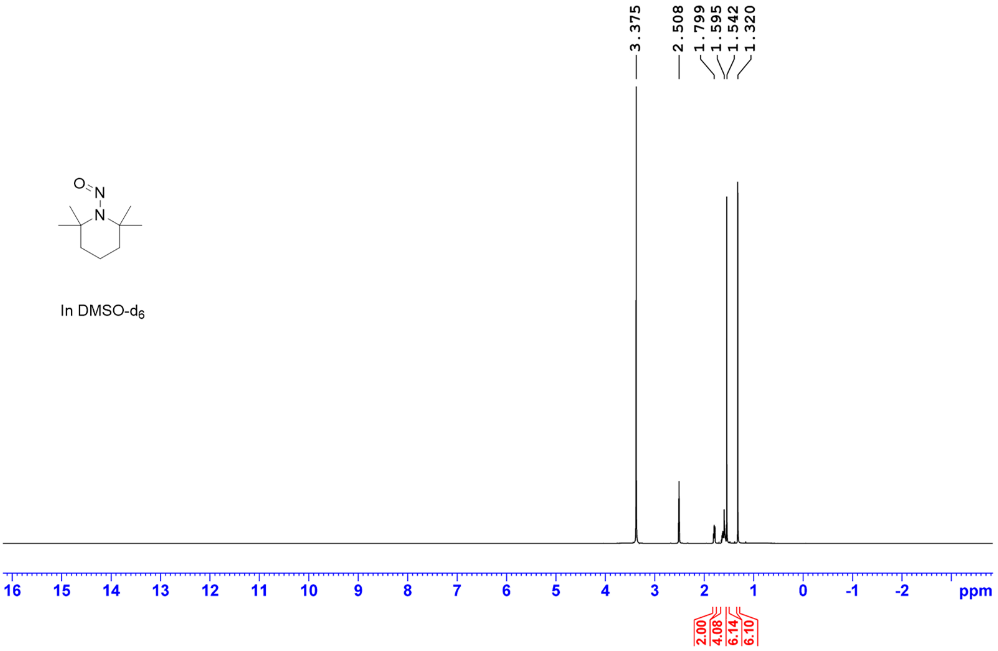
3.375
2.508
1.799
1.595
1.542
1.320
In DMSO-d6
16
15
14
13
12
11
10
9
8
7
6
5
4
3
2
1
0
-1
-2
ppm
2.00
4.08
6.14
6.10

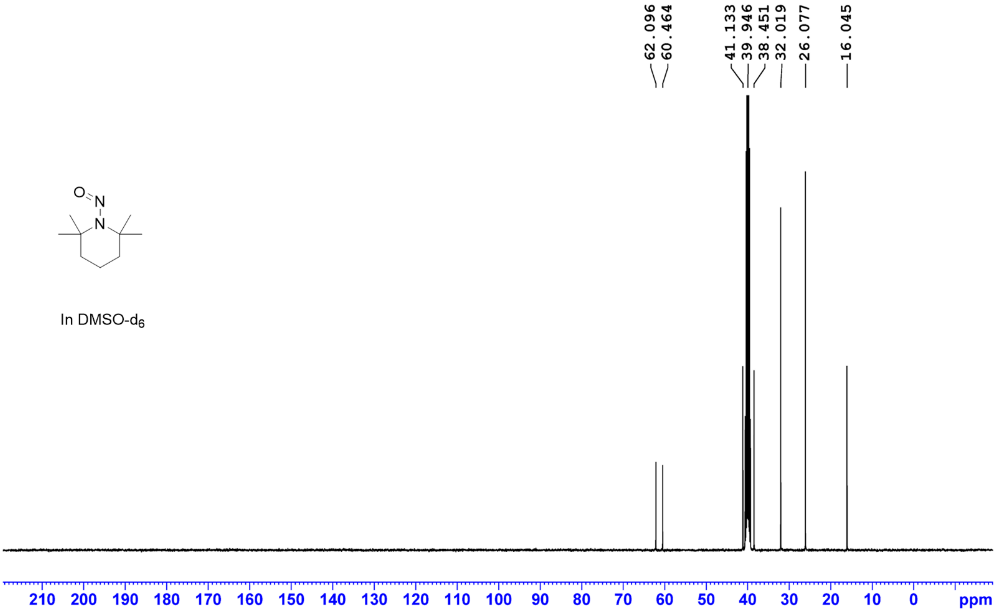
62.096
60.464
41.133
39.946
38.451
32.019
26.077
16.045
In DMSO-d6
210
200
190
180
170
160
150
140
130
120
110
100
90
80
70
60
50
40
30
20
10
0
ppm

References

1. Nishino, M.; Hirano, K.; Satoh, T.; Miura, M., Copper-Mediated and Copper-Catalyzed Cross-Coupling of Indoles and 1,3-Azoles: Double C=H Activation. *Angew. Chem. Int. Ed.* **2012**, *51*, 6993-6997.
2. Zhou, B.; Hu, Y.; Wang, C., Manganese-Catalyzed Direct Nucleophilic C(sp^2)–H Addition to Aldehydes and Nitriles. *Angew. Chem. Int. Ed.* **2015**, *54*, 13659-13663.
3. Liu, M.; Zhang, Z.; Song, J.; Liu, S.; Liu, H.; Han, B., Nitrogen Dioxide Catalyzed Aerobic Oxidative Cleavage of C(OH)–C Bonds of Secondary Alcohols to Produce Acids. *Angew. Chem. Int. Ed.* **2019**, *58*, 17393-17398.
4. Samanipour, M.; Ching, H. Y. V.; Sterckx, H.; Maes, B. U. W.; Van Doorslaer, S., The Non-innocent Role of Spin Traps in Monitoring Radical Formation in Copper-Catalyzed Reactions. *Appl. Magn. Reson.* **2020**, *51*, 1529-1542.
5. Bonke, S. A.; Risse, T.; Schnegg, A.; Brückner, A., In Situ Electron Paramagnetic Resonance Spectroscopy for Catalysis. *Nat. Rev. Dis. Primers* **2021**, *1*, 33.
6. Shimizu, M.; Watanabe, Y.; Orita, H.; Hayakawa, T.; Takehira, K., The Oxidation of 2,4,6-Trimethylphenol with Molecular Oxygen Catalyzed by a Copper(II)-Oxime or Copper(II)-Amine System. *Bull. Chem. Soc. Jpn.* **1993**, *66*, 251-257.
7. Chen, X.; Hao, X.-S.; Goodhue, C. E.; Yu, J.-Q., Cu(II)-Catalyzed Functionalizations of Aryl C−H Bonds Using O_2 as an Oxidant. *J. Am. Chem. Soc.* **2006**, *128*, 6790-6791.
8. Sambiagio, C.; Schönbauer, D.; Blieck, R.; Dao-Huy, T.; Pototschnig, G.; Schaaf, P.; Wiesinger, T.; Zia, M. F.; Wencel-Delord, J.; Besset, T.; Maes, B. U. W.; Schnürch, M., A Comprehensive Overview of Directing Groups Applied in Metal-Catalysed C–H Functionalisation Chemistry. *Chem. Soc. Rev.* **2018**, *47*, 6603-6743.
9. Yang, L.; Lu, Z.; Stahl, S. S., Regioselective Copper-Catalyzed Chlorination and Bromination of Arenes with O_2 as The Oxidant. *Commun. Chem.* **2009**, 6460-6462.
10. Song, S.; Sun, X.; Li, X.; Yuan, Y.; Jiao, N., Efficient and Practical Oxidative Bromination and Iodination of Arenes and Heteroarenes with DMSO and Hydrogen Halide: A Mild Protocol for Late-Stage Functionalization. *Org. Lett.* **2015**, *17*, 2886-2889.
11. Zhang, L.; Hu, X., Room Temperature C(sp^2)–H Oxidative Chlorination via Photoredox Catalysis. *Chem. Sci.* **2017**, *8*, 7009-7013.
12. Song, S.; Li, X.; Wei, J.; Wang, W.; Zhang, Y.; Ai, L.; Zhu, Y.; Shi, X.; Zhang, X.; Jiao, N., DMSO-Catalysed Late-Stage Chlorination of (hetero)Arenes. *Nat. Catal.* **2020**, *3*, 107-115.
13. Zhang, J.; Liu, J.-F.; Ugrinov, A.; Pillai, A. F. X.; Sun, Z.-M.; Zhao, P., Methoxy-Directed Aryl-to-Aryl 1,3-Rhodium Migration. *J. Am. Chem. Soc.* **2013**, *135*, 17270-17273.
14. Romanov-Michailidis, F.; Ravetz, B. D.; Paley, D. W.; Rovis, T., Ir(III)-Catalyzed Carbocarbation of Alkynes through Undirected Double C–H Bond Activation of Anisoles. *J. Am. Chem. Soc.* **2018**, *140*, 5370-5374.
15. Wu, Z.; Jiang, H.; Zhang, Y., Pd-catalyzed Cross-electrophile Coupling/C–H Alkylation Reaction Enabled by a Mediator Generated via C(sp^3)–H Activation. *Chem. Sci.* **2021**, *12*, 8531-8536.
16. Wu, Z.; Wei, F.; Wan, B.; Zhang, Y., Pd-Catalyzed ipso,meta-Dimethylation of ortho-Substituted Iodoarenes via a Base-Controlled C–H Activation Cascade with Dimethyl Carbonate as the Methyl Source. *J. Am. Chem. Soc.* **2021**, *143*, 4524-4530.
17. Xu, S.-M.; Chen, J.-Q.; Liu, D.; Bao, Y.; Liang, Y.-M.; Xu, P.-F., Aroyl Chlorides as Novel Acyl Radical Precursors via Visible-light Photoredox Catalysis. *Org. Chem. Front.* **2017**, *4*, 1331-1335.
18. Liu, Y.; Chen, Z.; Wang, Q.-L.; Chen, P.; Xie, J.; Xiong, B.-Q.; Zhang, P.-L.; Tang, K.-W., Visible Light-Catalyzed Cascade Radical Cyclization of N-Propargylindoles with Acyl Chlorides for the Synthesis of 2-Acyl-9*H*-pyrrolo[1,2-a]indoles. *J. Org. Chem.* **2020**, *85*, 2385-2394.
